# Supplementary material for: Do generational diversity and perceived similarity improve team functioning in rural Chinese hospitals? A cross-sectional survey study
Source: BMJ Open. 2024 Nov 27;14(11):e086451. doi: 10.1136/bmjopen-2024-086451 (PMC12207219; doi:10.1136/bmjopen-2024-086451)
Supplement: Supplementary data [file bmjopen-14-11-s001.pdf]

Appendix 1. Measures for perceived similarity, speaking up, silence and knowledge sharing

***Perceived similarity*[20,60]**

1. Team members handle problems in a similar way.

2. Team members think alike in terms of coming up with a similar solution.

3. Team members analyse problems in a similar way.

4. Team members see things in much the same way.

5. Team members are similar in terms of outlook and values.

6. Team members are alike in a number of areas.

***Speaking up*[61,62]**

1. I develop and make recommendations concerning issues that affect this team.

2. I speak up and encourage others in this team to get involved in issues that affect this team.

3. I communicate my opinions about work issues to others in this team even if my opinion is different and

4. I keep well informed about issues where my opinion might be useful to this team.

5. I get involved in issues that affect the quality of work life here in this team.

6. I speak up in this team with ideas for new projects or changes in procedures.

***Silence*[63-65]**

1. I withhold ideas from the team leader for changing inefficient work policies.

2. I keep ideas for developing new products or services to myself.

3. I do not speak up about difficulties caused by the way the team leader and the team members interact.

4. I keep quiet in team meetings about problems with daily routines that hamper performance.

5. I withhold thoughts about improving patients' experiences with us.

***Knowledge sharing*[66,67]**

1. Team members share information that can be helpful to the team.

2. Team members keep information flow high to increase team effectiveness.

3. Team members seek helpful information to be shared within the team.

4. Team members share expertise to help resolve the team’s problems.

5. Team members collectively offer innovative ideas that can benefit the team.
