# Supplementary material for: Do generational diversity and perceived similarity improve team functioning in rural Chinese hospitals? A cross-sectional survey study
Source: BMJ Open. 2024 Nov 27;14(11):e086451. doi: 10.1136/bmjopen-2024-086451 (PMC12207219; doi:10.1136/bmjopen-2024-086451)
Supplement: Supplementary data [file bmjopen-14-11-s002.pdf]

Appendix 2. *Additional multilevel mediation analysis (Diversity in the composition of local and non-local healthcare professionals)*

	Perceived similarity	Speak up	Silence	Knowledge sharing
Fixed effects				
**Within-group (Level 1)**				
Intercept	5.54**	1.76**	1.62**	2.39**
**Perceived similarity**	-	0.56**	0.39**	0.54**
Gender	0.23	0.04	-0.31	0.30**
Team tenure	-0.00	0.00	0.01	-0.01
**Between-group (Level 2)**				
**Diversity in composition**	-0.22	0.08	0.50	0.16
**Perceived similarity**	-	0.70**	0.40**	0.60**
Gender	0.25	-0.14	0.15	0.16
Team tenure	0.01	0.01	0.03	0.00
Team size	-0.00	-0.00	-0.00	-0.00
**Indirect effect (Mediation)**	-	-0.16	-0.09	-0.14
Random effects (Variance)				
Intercept	0.19**	0.03	0.40**	0.04
Slope	-	0.12**	0.04	0.11**

**: p<0.05; **: p<0.01*
