# Supplementary material for: Do generational diversity and perceived similarity improve team functioning in rural Chinese hospitals? A cross-sectional survey study
Source: BMJ Open. 2024 Nov 27;14(11):e086451. doi: 10.1136/bmjopen-2024-086451 (PMC12207219; doi:10.1136/bmjopen-2024-086451)
Supplement: Author's manuscript [file bmjopen-2024-086451.draft_revisions.pdf]

BMJ Open

BMJ Open is committed to open peer review. As part of this commitment we make the peer review history of every article we publish publicly available.

When an article is published we post the peer reviewers' comments and the authors' responses online. We also post the versions of the paper that were used during peer review. These are the versions that the peer review comments apply to.

The versions of the paper that follow are the versions that were submitted during the peer review process. They are not the versions of record or the final published versions. They should not be cited or distributed as the published version of this manuscript.

BMJ Open is an open access journal and the full, final, typeset and author-corrected version of record of the manuscript is available on our site with no access controls, subscription charges or pay-per-view fees (http://bmjopen.bmj.com).

If you have any questions on BMJ Open's open peer review process please email info.bmjopen@bmj.com

BMJ Open

# Healthcare workforce strengthening: Does (generational) diversity improve team functioning in rural Chinese hospitals? A cross-sectional study



| | |
|---|---|
| Journal: | *BMJ Open* |
| Manuscript ID | bmjopen-2024-086451 |
| Article Type: | Original research |
| Date Submitted by the Author: | 14-Mar-2024 |





| | |
|---|---|
| Complete List of Authors: | Wang, Hujie; Erasmus University Rotterdam<br>Buljac-Samardžić, Martina; Erasmus Universiteit Rotterdam<br>Van Wijngaarden, Jeroen; Erasmus Universiteit Rotterdam, Erasmus School of Health Policy & Management<br>van de Klundert, Joris; Erasmus University Rotterdam; Universidad Adolfo Ibáñez |





| | |
|---|---|
| Keywords: | Health Services, Human resource management < HEALTH SERVICES ADMINISTRATION & MANAGEMENT, Health policy < HEALTH SERVICES ADMINISTRATION & MANAGEMENT, Health Workforce |



SCHOLARONE™
Manuscripts

*I, the Submitting Author has the right to grant and does grant on behalf of all authors of the Work (as defined in the below author licence), an exclusive licence and/or a non-exclusive licence for contributions from authors who are: i) UK Crown employees; ii) where BMJ has agreed a CC-BY licence shall apply, and/or iii) in accordance with the terms applicable for US Federal Government officers or employees acting as part of their official duties; on a worldwide, perpetual, irrevocable, royalty-free basis to BMJ Publishing Group Ltd ("BMJ") its licensees and where the relevant Journal is co-owned by BMJ to the co-owners of the Journal, to publish the Work in this journal and any other BMJ products and to exploit all rights, as set out in our licence.*

*The Submitting Author accepts and understands that any supply made under these terms is made by BMJ to the Submitting Author unless you are acting as an employee on behalf of your employer or a postgraduate student of an affiliated institution which is paying any applicable article publishing charge ("APC") for Open Access articles. Where the Submitting Author wishes to make the Work available on an Open Access basis (and intends to pay the relevant APC), the terms of reuse of such Open Access shall be governed by a Creative Commons licence – details of these licences and which Creative Commons licence will apply to this Work are set out in our licence referred to above.*

*Other than as permitted in any relevant BMJ Author's Self Archiving Policies, I confirm this Work has not been accepted for publication elsewhere, is not being considered for publication elsewhere and does not duplicate material already published. I confirm all authors consent to publication of this Work and authorise the granting of this licence.*

For peer review only

# Healthcare workforce strengthening: Does (generational) diversity improve team functioning in rural Chinese hospitals? A cross-sectional study

**Hujie Wang[1*], Martina Buljac-Samardzic[1], Jeroen van Wijngaarden[1], Joris van de Klundert[1,2]**

[1] Erasmus School of Health Policy & Management, Erasmus University Rotterdam, Rotterdam, the Netherlands
[2] School of Business, Universidad Adolfo Ibáñez, Santiago, Chile
* **Correspondence**: Hujie Wang (wang@eshpm.eur.nl)

## Abstract

**Introduction**: Hospitals in developing countries have begun to increase the recruitment of healthcare workforce which is recommended by the United Nations to achieve universal health coverage. Generational diversity, increasingly prominent in the composition of healthcare workforce in rapidly developing countries, has received much attention in practice and research recently. While research has revealed various positive and negative impacts of generational diversity on team functioning, the understanding of the mechanism explaining how generational diversity influences team functioning is still limited. This study in rural Chinese hospitals examines the relationship between (surface-level) generational diversity and (deep-level) perceived similarity and investigates how they influence three teamwork behaviours that importantly determine quality of care: speaking up, silence and knowledge sharing.

**Methods**: Via an online survey, 841 healthcare professionals from four rural Chinese hospitals gave their views on generational diversity, perceived similarity, speaking up, silence and knowledge sharing. Multilevel mediation hypotheses were tested.

**Results**: Perceived similarity is positively related to the three teamwork behaviours, i.e. speaking up, silence and knowledge sharing, while generational diversity is not. As the relationship between generational diversity and perceived similarity is non-significant, perceived similarity does not mediate the relationship between generational diversity and teamwork behaviour.

**Conclusion**: This study in rural Chinese hospitals confirms the positive relationship between the deep-level perceived similarity, and the three team behaviours speaking up, silence and knowledge sharing, and finds that generational diversity is not significantly related with perceived similarity nor with these three team behaviours. This suggests that increases in generational diversity that results from healthcare workforce strengthening may not impact team behaviours and performance. However, explicit management efforts to mitigate the negative impact on team behaviour and care provision are called for if the healthcare workforce strengthening required for achieving universal health coverage reduces perceived similarity in teams.

***Keywords***: generational diversity, perceived similarity, teamwork, speaking up, silence, knowledge sharing

**Strengths and limitations of this study**



For peer review only - http://bmjopen.bmj.com/site/about/guidelines.xhtml

- Multilevel analysis was used to provide more accurate insights into the relationships among the variables by eliminating the cluster effects of the data.
- Cross-sectional design does not allow to claim causal relationships.
- Using one method to collect data from the same respondents may produce common method bias and common resource bias.

## Introduction

Sustainable Development Goal 3 aims at good health and well-being for all and at universal health coverage (UHC) as a subgoal towards achieving this aim.[1] Many developing countries experience barriers to providing high quality care, a key component of UHC, because of healthcare workforce shortages. These shortages are particularly severe in rural and remote areas.[2-4] Improvement of healthcare workforce management in developing countries, including recruitment, development and retention, can form an effective strategy towards UHC.[1] Attracting and retaining more highly qualified workforce often requires entertaining a greater variety of healthcare professionals, thus increasing the diversity in the workforce and healthcare teams. This diversity may entail a wider variety of generations, professionals and cultural or geographical backgrounds.

Healthcare workforce diversity can impact healthcare team performance both positively (e.g. better health outcomes) and negatively (e.g. increased conflict).[5,6] However, the evidence base is predominantly from developed countries,[5,6] leaving the role of workforce diversity in the performance of rural healthcare teams in developing countries where UHC is most difficult to achieve under-researched.

This study focuses on diversity of healthcare teams and emphasises generational diversity. Successfully engaging multiple generations in healthcare teams forms an increasingly common and important pathway to address workforce shortages and provide high quality care.[7] The evidence on the relationship between generational diversity and team functioning is scarce, inconclusive and mostly from developed countries.[8,9] For example, generational diversity can promote innovative team behaviour yet may also produce communication barriers.[10,11] We aim to expand the evidence base on the relationship between diversity, particularly generational diversity, and healthcare team functioning in rural areas of developing countries and conducted a study involving four rural Chinese hospitals.

In recent decades, many developing countries have experienced rapid and profound economic developments and social changes, which cause larger generational differences in these countries compared to developed countries with more modest development rates. For instance, China has achieved an average yearly GDP growth rate of 9.1% since initiating social reforms and opening-up in 1978, nearly threefold the 3.1% yearly growth of the global GDP during the same period.[12] Such dramatic economic developments and social changes can enlarge the differences in behaviours and values between generations,[13] where a generation is defined as a group of people sharing birth years in a certain period and raised in a similar social and development stage.[14,15] These differences in behaviours and values may subsequently influence team functioning and performance.

Rural Chinese hospitals have been reported to traditionally employ a less diverse workforce, to recruit mostly from the local population, and to have difficulties in attracting highly educated healthcare professionals.[16] For these rural hospitals, which provide health services for nearly half a billion rural Chinese citizens,[17] recruitment of young, highly educated and nonlocal professionals forms a (generational) diversity increasing pathway towards delivering high quality care envisioned



For peer review only - http://bmjopen.bmj.com/site/about/guidelines.xhtml

to be part of UHC.[1] To the best of our knowledge, existing evidence of the challenges posed by (generational) diversity on team functioning in rural healthcare in China and elsewhere is scarce and mostly of a qualitative nature.[2,16,18] This study in rural China presents a first quantitative study into the relationship between diversity and teamwork behaviour.

Generational diversity is based on demographic characteristics and therefore regarded as a form of surface-level diversity.[19,20] Generational diversity may lead to perceived (dis)similarities between generations regarding communication styles and work attitudes.[21,22] Such perceived (dis)similarities are forms of deep-level diversity, as they are associated with underlying attributes such as beliefs, attitudes and values.[20] Jansen & Searle's review on team diversity recommends researching surface-level and deep-level diversity simultaneously.[23]

Empirical evidence and several well-known theories support a relationship between perceived similarity and team functioning. Shemla et al.'s review reports that perceived similarity is positively related to team members' supportive behaviour (i.e. social and task exchange) and team commitment and negatively related to employees' intention to resign.[24] Jansen & Searle's review also states that perceived similarity benefits team performance, team efficiency and job satisfaction.[23] The similarity attraction theory[25] suggests that similar team members understand each other's thoughts and behaviours better and, therefore, are more likely to interact with one another.[20,26] In addition, the social identity theory[27] and the self-categorisation theory[28] propose that team members may categorise other team members they perceived to be similar to themselves as "ingroups" with whom they are more willing to interact than with less similar team members, the "outgroups".

As perceived similarity may be directly associated with communication and interaction, we operationalise team functioning through three individual behaviours which are especially considered to be essential for healthcare delivery: speaking up, silence and knowledge sharing. Silence and speaking up refer to the extent individuals voice work-related issues. Several reviews and studies present the positive effect of speaking up[29,30] and the negative effect of silence[31] on team functioning in healthcare. Over the past years, knowledge sharing has also received much attention in healthcare.[32-34] Team members might still share knowledge to promote team functioning even when the team climate does not support speaking up and/or speaking up is not seen as appropriate.

In case generational diversity translates into perceived similarity, and the latter impacts speaking up, silence and knowledge sharing, perceived similarity acts as a mediating mechanism via which generational diversity impacts the three teamwork behaviours. This study aims to investigate how generational diversity and perceived similarity influence speaking up, silence and knowledge sharing in rural Chinese hospitals.

## Hypotheses

### *Relationship between generational diversity and perceived similarity*

Generational diversity arises when a team includes members from at least two generations. Commonly considered generations in scientific literature are Baby Boomer (born between 1946 and 1964), Generation X (born between 1965 and 1981) and Generation Y (born between 1982 and 2000).[35] This categorisation is rooted in Western context and evidence and may not have external validity in rural China which has developed quite differently. In this study, we follow the generational categorisation mostly used in China-related literature which distinguishes the decades "1960s,



For peer review only - http://bmjopen.bmj.com/site/about/guidelines.xhtml

1970s, …" to define corresponding generations.[13,36-38] Each of these generations has its characteristics that are relevant for team functioning. For example, in China, people born in the 1970s are more collectivist and pragmatic, while those born in the 1980s are more individualistic and confident.[13,37]

Perceived similarity is one's perception of the similarity between people[39] and is related to deep-level attributes (i.e. underlying elements such as values, attitudes and beliefs).[20] (Surface-level) generational diversity may underpin perceived similarity via generational differences in values, attitudes and beliefs.[8,40] In a generationally diverse team, people from different generations may therefore hold distinct values, attitudes and beliefs and will, therefore, perceive those from other generations dissimilar to themselves. For the same reason, team members may be more likely to consider other team members from the same generation as similar to themselves. We thus propose:

**Hypothesis 1**: Generational diversity is negatively related to perceived similarity.

### *Relationship between perceived similarity and teamwork behaviours*

Speaking up, also known as voice behaviour, is defined as expressing one's opinions about any work-related matters.[41,42] Employee silence refers to "withholding ideas, information and opinions" relevant to the improvements in workplace.[43] Speaking up and silence are not affirmatively opposite and can coexist. Individuals who speak up on certain issues can intentionally withhold their ideas and keep silent about other issues at the same time.[43,44] Knoll & Redman even find that some types of speaking up and silence behaviours (i.e. promotive speaking up and cooperative silence) are positively correlated.[45]

Sometimes, team members may not be able or willing to point out work-related issues but may still practice knowledge sharing. Knowledge sharing refers to people sharing their work-related knowledge with team members to help improve the team effectiveness.[46,47]

Antecedents of speaking up, silence and knowledge sharing are identified by previous reviews and studies. For example, some reviews summarise the factors influencing speaking up and silence behaviour (e.g., motivation, contextual factors, individual factors and organisational factors).[30,48-50] S. Wang & Noe have studied the factors influencing knowledge sharing and provide evidence that diversity on demographic features (e.g., gender, education and marital status) is associated with knowledge sharing in non-healthcare settings.[51] Workplace ostracism has been shown to be negatively related to speaking up[52,53] and knowledge sharing[54.55] but to be positively associated with silence[53,56] and knowledge hiding.[57,58]

The similarity attraction theory suggests that people will be attracted by those similar to themselves but ostracise dissimilar ones.[25] According to this theory, individuals more easily build good interpersonal relations with similar ones, understand the thoughts and behaviours of similar ones and are subsequently more likely to interact with them.[20,26] Following this line of reasoning, team members would be more likely to speak up and share knowledge within the team and less likely to withhold their ideas if they perceive themselves more similar to each other. Conversely, team members would be more reluctant to express their ideas and share their knowledge and more prone to keeping silent with team members perceived as dissimilar from themselves. Therefore, we propose:

**Hypothesis 2a**: Perceived similarity is positively related to speaking up.



For peer review only - http://bmjopen.bmj.com/site/about/guidelines.xhtml

**Hypothesis 2b**: Perceived similarity is negatively related to silence.

**Hypothesis 2c**: Perceived similarity is positively related to knowledge sharing.

### *The mediating role of perceived similarity*

The similarity attraction theory posits that individuals' actual similarity (e.g., demographic characteristics) leads to perceived similarity and consequently influence people's behaviour.[25] As generational diversity creates dissimilarity, individuals may perceive team members from other generations to be dissimilar, causing them to be less likely to speak up, more likely to remain silent and less likely to share knowledge. Likewise, low generational diversity within teams, as occurs when there are few generational differences between team members, would imply high perceived similarity and to promote the speaking up and knowledge sharing and reduce silence. We accordingly propose:

**Hypothesis 3**: Perceived similarity mediates the relationship between generational diversity and a) speaking up, b) silence and c) knowledge sharing.

## Methods

### *Sample and procedure*

Following our research aims, we approached rural hospitals in China and more specifically seven county-level hospitals, with the help of the Health Human Resources Development Centre of the National Health Commission of China and the County Health Media. The former one is responsible for promoting the human resource management for hospitals of all levels in China, while the latter one is a media company focusing on China's county-level hospitals. Four out of the seven county-level hospitals accepted our invitations to participate in the study. These four hospitals are located in four different provincial administrative regions across China. Together, these four hospitals employ 3500 employees, among whom are around 1000 doctors and 1700 nurses.

This study was conducted in the form of an online survey that was disseminated via the widely used Chinese survey platform "Wen Juan Xing" in October 2022. Participants were team members, including doctors, nurses and other healthcare professionals, working in monodisciplinary teams from the four county-level hospitals. Team leaders were only included for the measurement of generational diversity but not for the other constructs.

The Research Ethics Review Committee of Erasmus School of Health Policy and Management, Erasmus University Rotterdam approved this study (No. ETH2122-0807). Consent was obtained from each respondent before the data collection.

### *Measures*

***Generational diversity***. Following existing literature on the definition of generations in China, we specified the generations into 5 categories: born between 1960 and 1969, born between 1970 and 1979, born between 1980 and 1989, born between 1990 and 1999, and born after 2000.[13,36-38] Team members and team leaders were asked to fill in the number of people for each category on their teams. The generational diversity perceived by each participant is calculated using Blau's index[59]:



For peer review only - http://bmjopen.bmj.com/site/about/guidelines.xhtml

$1 - \sum_{i=1}^{n} p_i^2$, where $p_i$ is the perceived proportion of team members in the $i^{th}$ category (generation), and $n$ is the perceived number of categories (generations) in the team. Blau's index equals 0 if all team members are from the same generation and increases as the members are divided over more generations. As generational diversity is a team-level construct, we calculated the mean of the individual Blau's indices of team members and team leaders for each team to express perceived generational diversity at the team-level.

***Perceived similarity***. We measured the perceived similarity between team members using Williams et al.'s 6-item measure,[20] which was adapted from Liden et al.[60] This scale is a 7-point Likert scale, ranging from 1 (strongly disagree) to 7 (strongly agree). The Cronbach's α in this study is 0.97.

***Speaking up***. Speaking up was measured by a 6-item measure, developed by van Dyne and LePine[61] and adapted by Morrison et al.[62] This 7-point Likert scale ranges from 1 (strongly disagree) to 7 (strongly agree). The Cronbach's α in this study is 0.94.

***Silence***. A 5-item measure developed by Detert and Edmonson[63] and adapted by Guenter et al.[64] and Mignonac et al.[65] was used to test employee silence. The items were rated on a 7-point Likert scale ranging from 1 (strongly disagree) to 7 (strongly agree). The Cronbach's α in this study is 0.94.

***Knowledge sharing***. We assessed knowledge sharing with Pittino et al.'s 5-item measure,[66] as adapted from Bartol et al.[67] A 7-point Likert scale was used for rating, ranging from 1 (strongly disagree) to 7 (strongly agree). The Cronbach's α in this study is 0.99.

***Control variables***. From a wide set of literature based control variables, gender and team tenure were significantly associated with one of the dependent variables in preliminary analysis and therefore included.[20,61,62,64,67,68] Furthermore, we also controlled for team size at team level as it is found to influence speaking up and other interaction within a team in previous research.[69,70]

The measures for perceived similarity, speaking up, silence and knowledge sharing are presented in Appendix 1. All measures were translated from English to Chinese using the standard translation/back-translation technique before data collection.[71] The average scores of the items for each scale were used to form the individual measurements of perceived similarity, speaking up, silence and knowledge sharing for each participant.

### *Analysis*

All the analyses were conducted via SPSS 29 and AMOS 28. We conducted descriptive and correlation analyses via SPSS and confirmatory factor analysis (CFA) via AMOS to provide an overview of the data. The CFA shows good factor loadings for perceived similarity, speaking up, silence and knowledge sharing, ranging from 0.72 to 0.99. The model fit indices of the CFA suggest that the four-factor model (i.e., a model with perceived similarity, speaking up, silence and knowledge sharing being separate factors) presents an acceptable model fit ($\chi^2(203)=1796.92$, CFI=0.94, TLI=0.94, RMSEA=0.10, SRMR=0.05) and fits significantly better than the three-factor model (i.e. combining speaking up and silence into one factor; $\chi^2(206)=5491.54$, $p<0.01$, CFI=0.81, TLI=0.79, RMSEA=0.18, SRMR=0.14) , two-factor model (i.e. combining speaking up, silence and knowledge sharing; $\chi^2(208)=8163.26$, $p<0.01$, CFI=0.71, TLI=0.68, RMSEA=0.21, SRMR=0.18) and one-factor model (i.e. combining all the four as one factor; $\chi^2(209)=12430.70$, $p<0.01$, CFI=0.56, TLI=0.51, RMSEA=0.26, SRMR=0.21).



For peer review only - http://bmjopen.bmj.com/site/about/guidelines.xhtml

Multilevel analysis was used to analyse the data as respondents were nested within teams, and teams were further nested within hospitals, which caused dependencies between observations. The standard mixed linear models in SPSS showed that there were significant between-team variances for the three dependent variables (i.e. speaking up, silence and knowledge sharing), indicating the necessity to conduct a multilevel analysis. However, between-hospital variances were not significant. Therefore, a two-level mediation analysis was performed; within-group (level 1) was the individual level, while between-group (level 2) was the team level. We chose the random effects model for the multilevel mediation analysis as it is expected that the intercepts and slopes will vary across higher-level groups (i.e. teams).[72]

We used the MLmed macro in SPSS to conduct the multilevel mediation analysis.[73] In the interface of the MLmed macro, three different random effects models were built with "speaking up", "silence" and "knowledge sharing" (level 1) as dependent variables. "Generational diversity" (level 2) served as the independent variable, while "perceived similarity" (level 1) served as a mediator. These three models were 2-1-1 multilevel mediation models in which the independent variable was a team-level (level 2) variable, and the other variables were at the individual level (level 1). The control variables gender and team tenure were level-1 covariates, and team size was a level-2 covariate.

As generational diversity is a level-2 variable, the random-effect parameters only account for the intercepts of the mediator and dependent variables and the slopes regarding the relationships between perceived similarity and the three behavioural variables (i.e., speaking up, silence and knowledge sharing) as these four variables are all at level 1.

Additional multilevel mediation analyses replacing generational diversity by the diversity in the composition of local and non-local healthcare professionals were conducted to provide more insights for future research. The reporting of this study follows the Strengthening the Reporting of Observational Studies in Epidemiology Statement (STROBE).[74]

### *Patient and public involvement*

Patients or the public were not involved in design, conduct, reporting or dissemination plans of our research.

## Results

### *Descriptive analysis*

841 valid questionnaires were received. The average age of the 841 respondents is 32.10 years (median: 31.00; standard deviation: 8.08). The average team tenure is 6.71 years (median: 5.00; standard deviation: 6.37). The percentage of doctors in the respondents (38.64%) is similar to that in all Chinese hospitals (38.04%), while the percentage of nurses (56.96%) is higher than the national data (44.81%).[75] The respondents' demographic characteristics are shown in Table 1.

*Table 1. Demographic characteristics*

| | **Number of people (Percentage)** |
|---|---|
| **Gender** | |
| Male | 165 (19.62%) |



For peer review only - http://bmjopen.bmj.com/site/about/guidelines.xhtml

| | |
|---|---|
| Female | 652 (77.53%) |
| Prefer not to say | 24 (2.85%) |
| **Age*** | |
| <=30 | 414 (49.23%) |
| 31-40 | 287 (34.13%) |
| 41-50 | 103 (12.25%) |
| >=51 | 32 (3.80%) |
| **Profession** | |
| Doctors | 325 (38.64%) |
| Nurses | 479 (56.96%) |
| Other healthcare professionals | 37 (4.40%) |
| **Local or non-local** | |
| Local | 731 (86.92%) |
| Non-local | 110 (13.08%) |
| **Education background** | |
| Master | 18 (2.14%) |
| Bachelor | 593 (70.51%) |
| Lower than bachelor | 230 (27.35%) |
| **Professional title** | |
| Senior | 17 (2.02%) |
| Deputy senior | 55 (6.54%) |
| Intermediate | 215 (25.56%) |
| Junior | 554 (65.87%) |

** There are missing values regarding age, so the sum of the number of people per age group is smaller than the total number of respondents.*

The correlation analyses (Table 2) show significant strong correlations between on the one hand perceived similarity and on the other hand speaking up ($r$= 0.65, $p$<0.01) and knowledge sharing ($r$= 0.67, $p$<0.01) but a small to moderate correlation between perceived similarity and silence ($r$=0.26, $p$<0.01). Significant strong correlation is also shown between speaking up and knowledge sharing ($r$= 0.75, $p$<0.01), while the positive correlation between speaking up and silence is moderate ($r$=0.33, $p$<0.01). Generational diversity at team level is not significantly correlated to either perceived similarity or individual behaviours. Full collinearity test shows that the values of the variance inflation factors for all the control variables, independent variable and mediator range from 1.01 to 1.12, indicating no multicollinearity issues in this study.[76]

*Table 2. The correlation matrix of all variables*

| | 1 | 2 | 3 | 4 | 5 | 6 | 7 | 8 |
|---|---|---|---|---|---|---|---|---|
| 1. Gender (1=female) | 1.00 | | | | | | | |
| 2. Team tenure | -0.19** | 1.00 | | | | | | |
| 3. Team size | 0.02 | 0.10** | 1.00 | | | | | |
| 4. Generational diversity*** | -0.14** | 0.23** | 0.22** | 1.00 | | | | |
| 5. Perceived similarity | 0.07 | 0.00 | -0.03 | -0.00 | 1.00 | | | |
| 6. Speaking up | 0.02 | 0.04 | -0.06 | 0.01 | 0.65** | 1.00 | | |
| 7. Silence | -0.02 | 0.06 | -0.01 | 0.04 | 0.26** | 0.33** | 1.00 | |
| 8. Knowledge sharing | 0.12** | -0.01 | -0.03 | 0.00 | 0.67** | 0.75** | 0.15** | 1.00 |



For peer review only - http://bmjopen.bmj.com/site/about/guidelines.xhtml

***: p<0.01*

****: The generational diversity is a team-level construct, and thus a single value is assigned to all the individuals from the same team within the range of 0 and 0.8.*

***Multilevel mediation analysis***

The three 2-1-1 multilevel mediation models are shown in Table 3.

*Table 3. Multilevel mediation analyses*

| | Perceived similarity | Speaking up | Silence | Knowledge sharing |
|---|---|---|---|---|
| ***Fixed effects*** | | | | |
| **Within-group (Level 1)** | | | | |
| Intercept | 5.46** | 1.75** | 1.65* | 2.39** |
| **Perceived similarity** | - | 0.56** | 0.39** | 0.54** |
| Gender | 0.23 | 0.04 | -0.31 | 0.30** |
| Team tenure | -0.00 | 0.00 | 0.01 | -0.01 |
| **Between-group (Level 2)** | | | | |
| **Generational diversity** | 0.07 | 0.08 | 0.44 | 0.09 |
| **Perceived similarity** | - | 0.69** | 0.38** | 0.60** |
| Gender | 0.27 | -0.15 | 0.11 | 0.14 |
| Team tenure | 0.01 | 0.01 | 0.02 | 0.00 |
| Team size | -0.00 | -0.00 | -0.00 | -0.00 |
| **Indirect effect (Mediation)** | - | 0.05 | 0.03 | 0.04 |
| ***Random effects (Variance)*** | | | | |
| Intercept | 0.19** | 0.03 | 0.41** | 0.04 |
| Slope | - | 0.12** | 0.04 | 0.11** |

**: p<0.05; **: p<0.01*

Table 3 shows that there is no significant relationship between generational diversity and perceived similarity ($\beta$=-0.07, $p$>0.05). Therefore, hypothesis 1 is rejected, which also violates the first requirement for a mediating relationship.

The results also show that perceived similarity is positively related to all the three behavioural variables at both the within-group level (speaking up: $\beta$= 0.56, p<0.01; silence: $\beta$=0.39, p<0.01; knowledge sharing: $\beta$=0.54, p<0.01) and between-group level (speaking up: $\beta$=0.69, p<0.01; silence: $\beta$=0.38, p<0.01; knowledge sharing: $\beta$=0.60, p<0.01). These findings indicate that people will be more likely to speak up, keep silent and share knowledge when they perceive themselves more similar to team members. As a result, these results support hypotheses 2a and 2c but reject hypothesis 2b, which specifies a negative relationship between perceived similarity and silence.

The random-effect parameters show that the relationships between perceived similarity and speaking up (0.12, p<0.01) and between perceived similarity and knowledge sharing (0.11, p<0.01) significantly vary across groups, while the relationship between perceived similarity and silence (0.04, p>0.05) does not significantly change between groups. These findings illustrate that within the teams with the same extent of perceived similarity, team members in some of these teams are more willing to speak up and share knowledge than those in some other teams, but the extent of keeping



For peer review only - http://bmjopen.bmj.com/site/about/guidelines.xhtml

silent does not vary across teams. Furthermore, the intercepts of perceived similarity and silence vary across groups, while those of speaking up and knowledge sharing do not. These results indicate that team members from different teams have different degrees of the perceptions towards other team members and keeping silent. However, the extent of speaking up and sharing knowledge remain constant across teams.

As generational diversity is a level-2 variable, only between-group direct effects (i.e., the relationships between generational diversity and the three behavioural variables) and mediated effects (i.e., indirect effects) are evaluated. As shown in Table 3, there are neither significant direct effects of generational diversity on the three behavioural variables (speaking up: $\beta$=0.08, p>0.05; silence: $\beta$=0.44, p>0.05; knowledge sharing: $\beta$=0.09, p>0.05) nor significant mediated effects via perceived similarity (speaking up: $\beta$=0.05, p>0.05; silence: $\beta$=0.03, 0>0.05; knowledge sharing: $\beta$=0.04, p>0.05). Therefore, hypothesis 3 is not supported.

**Discussion**

This study investigates the relationships among generational diversity, perceived similarity, speaking up, silence and knowledge sharing in rural Chinese hospitals. In contrast to the theory-informed hypotheses formulated, this study finds no significant relationships between generational diversity and the other four variables (i.e., perceived similarity, speaking up, silence and knowledge sharing) and therefore appears to exclude significant direct and mediated effects of generational diversity on these variables.

Generational diversity is defined on the basis of age groups and therefore on a surface-level attribute.[19,20] Literature shows that surface-level similarity is not necessarily in alignment with perceived similarity.[20,77] We tried to establish the connection between generational diversity and perceived similarity via intergenerational differences as the latter two are related to deep-level attributes (e.g., values, attitudes and beliefs) but found non-significant relationship. Other determinants of perceived similarity exist as well, which may influence perceived similarity more strongly. For instance, Ott-Holland et al. demonstrate that gender plays a role in perceived similarity, and females perceive themselves more similar to others than do males.[78] H. Wang et al. find that local origin may impact perceived similarity.[16] People raised in the same place share the local culture, norms and social background and therefore are more likely to form similar values and beliefs. Of the respondents included in this study, 86.85% were born and raised in the areas where their hospitals were located. An additional analysis, however, reveals that the composition of local and non-local employees is not significantly related to perceived similarity, nor to individual behaviours (Appendix 2). More research is needed to understand the determinants of perceived similarity.

Our findings show that perceived similarity is positively associated with speaking up and knowledge sharing at both individual and team level. These findings confirm the similarity attraction theory, which posits such interactive behaviours to be associated with similarity.[25] The social identity theory[27] and the self-categorisation theory[28] provide further theoretical support for this finding. Similar people will categorise themselves as “ingroups”, enhance their self-image and then be more willing to interact with each other. Conversely, persons perceive dissimilar ones as “outgroups” and amplify the differences between “ingroups” and “outgroups”, which may form intergroup conflicts and finally reduce the frequency of intergroup interactive behaviours. The evidence found and theoretical support thus indicate that increasing the recruitment of healthcare workforce in rural



For peer review only - http://bmjopen.bmj.com/site/about/guidelines.xhtml

Chinese hospitals (and other rural settings) to strengthen the quality of care and UHC may negatively influence speaking up and knowledge sharing and impede teamwork if it causes perceived dissimilarity to increase.

Counter to our hypothesis, perceived similarity is positively related to silence at both individual and team level. The social identity theory[27] and the self-categorisation theory[28] may provide explanations for this finding. Team members may consider other team members perceived as similar as "ingroups" with whom they develop personal relationships. These personal relationships may subsequently lead them to refrain from commenting openly on the functioning of these "ingroups". As this study was conducted in rural China, the Chinese cultural value of "saving face" and "harmony" to promote team functioning may further amplify this mechanism.[79] This cultural perspective sheds a novel light on the common, perhaps Western, view that silence is harmful to team functioning in healthcare.[31] Furthermore, team members may be less likely to remain silent about or with dissimilar team members ("outgroups"), as perceived dissimilarity is always accompanied by dissatisfaction with others.[80,81]

In addition to the positive association between perceived similarity and both of speaking up and silence, we somewhat surprisingly find a positive correlation between the latter two. This correlation may be explained by distinguishing various subtypes of speaking up and silence. Scientific literature has identified acquiescent speaking up/silence (expressing/withholding ideas based on resignation and low self-efficacy), defensive speaking up/silence (expressing/withholding ideas based on self-protection and fear) and prosocial speaking up/silence (expressing/withholding ideas based on benefiting the organisation and cooperation).[43] These different forms of speaking up and silence can, for example, explain that team members express constructive suggestions with the goal of benefiting the team while keeping silent to protect themselves and their "ingroups" on other occasions. Our findings from the rural Chinese context thus confirm that speaking up and silence are distinct constructs rather than a pair of opposite behaviours.[43,44] It is worthwhile to investigate subtypes of speaking up and silence, and their relationship with perceived similarity in future research on team functioning and performance, especially in settings in which health workforce strengthening is likely to increase dissimilarity.

***Practical implications***

Our study shows teamwork behaviour is associated with perceived similarity yet less so with generational diversity. This is relevant as the teamwork behaviours studied (i.e. speaking up, silence and knowledge sharing) are associated with team performance and quality of care. Thus, rural Chinese hospital management may leverage the benefits of perceived similarity for team functioning to improve the quality of care and UHC. At the same time, our findings indicate that increases in generational diversity, which may result from ongoing workforce strengthening efforts, will not negatively impact the studied teamwork behaviours in support of healthcare quality and achieving UHC. However, when recruitment efforts for workforce strengthening lead to more perceived dissimilarity in teams, these dissimilarities need to be managed, for instance through team training, to avoid negative impacts on team functioning and, possibly, quality of care.

***Limitation***



For peer review only - http://bmjopen.bmj.com/site/about/guidelines.xhtml

There are several limitations for this study. First, only four Chinese county-level hospitals were included, which may lead to a selection bias and may not depict a full picture of all rural Chinese hospitals. Still, of the four participating hospitals, two are from distinct northern Chinese regions, and the other two from distinct southern Chinese regions. Second, the number of people who received the survey link is unknown, so we are not able to calculate the response rate. Third, all the variables were measured with the same respondents and via the same data collection method (survey), which may produce common source bias and common method bias. Fourth, the cross-sectional design does not allow claims about causality of the significant relationships between perceived similarity and the three teamwork behaviours studied.

## Conclusion

Perceived similarity is positively related to speaking up, silence and knowledge sharing at both individual and team level. Generational diversity has no significant relationships with perceived similarity nor with these three teamwork behaviours. Thus, if healthcare workforce strengthening increases generational diversity, this may not impact team behaviour and performance. However, explicit management efforts to mitigate the negative impact on team behaviour and care provision are called for if the healthcare workforce strengthening required for improving quality of care and UHC reduces perceived similarity in teams. More research is needed to explore the antecedents of (deep-level) perceived similarity and the interaction between perceived similarity and various forms of speaking up and silence.

## Author contribution

Study design: HW, MBS, JVW, JVDK.

Data collection: HW

Data analysis: HW, MBS.

Drafting the manuscript: HW.

Critical revisions of the manuscript: HW, MBS, JVW, JVDK.

Final approval of the manuscript: HW, MBS, JVW, JVDK.

## Acknowledgement

We thank Yun Zhang and Jing Zhou from the Health Human Resources Development Centre of the National Health Commission of China, and Yan'an Wang and Xiaoxiao Wan from the County Health Media for helping connect Chinese hospitals. We are also grateful for Wenxing Wang's participation in the translation/back-translation before data collection. Most importantly, we show our highest gratitude to the presidents and all the respondents from the 4 rural Chinese hospitals for their contributions to this study.

## Ethical statement



For peer review only - http://bmjopen.bmj.com/site/about/guidelines.xhtml

The Research Ethics Review Committee of Erasmus School of Health Policy and Management, Erasmus University Rotterdam approved this study (No. ETH2122-0807). Consent was obtained from each respondent before the data collection.

**Conflicts of interest**

The authors declare that this study was conducted without any conflicts of interest.

**Funding**

This study was supported by China Scholarship Council (Grand number: 201906160092). This funder has no role in the study design, data collection, data analysis and writing the manuscript.

**Data availability statement**

The data that support the findings of this study are available from the corresponding author upon reasonable request.

**References**

1. World Health Organization. Targets of Sustainable Development Goal. Available: https://www.who.int/data/gho/data/themes/sustainable-development-goals [Accessed 13 Feb 2024].

2. Lehmann U, Dieleman M, Martineau T. Staffing remote rural areas in middle-and low-income countries: a literature review of attraction and retention. *BMC Health Serv Res* 2008;**8**:19. doi:10.1186/1472-6963-8-19

3. Maffioli EM, Rocha TAH, Vivas G, *et al*. Addressing inequalities in medical workforce distribution: evidence from a quasi-experimental study in Brazil. *BMJ Glob Health* 2019;**4**:e001827. doi:10.1136/bmjgh-2019-001827

4. Sheikh K, Josyula LK, Zhang X, *et al*. Governing the mixed health workforce: learning from Asian experiences. *BMJ Glob Health* 2017;**2**:e000267. doi:10.1136/bmjgh-2016-000267

5. Gomez LE, Bernet P. Diversity improves performance and outcomes. *J Natl Med Assoc* 2019;**111**:383-92. doi:10.1016/j.jnma.2019.01.006

6. Guglielminotti J, Samari G, Friedman AM, *et al*. Nurse workforce diversity and reduced risk of severe adverse maternal outcomes. *Am J Obstet Gynecol MFM* 2022;**4**:100689. doi:10.1016/j.ajogmf.2022.100689

7. Spinks N, Moore C. The changing workforce, workplace and nature of work: implications for health human resource management. *Nurs Leadersh* 2007;**20**:26-41. doi:10.12927/cjnl.2007.19286

8. Lyons S, Kuron L. Generational differences in the workplace: a review of the evidence and directions for future research. *J Organ Behav* 2014;**35**:S139-57. doi: 10.1002/job.1913



For peer review only - http://bmjopen.bmj.com/site/about/guidelines.xhtml

9. Woodward I, Vongswasdi P, More E. Generational Diversity at Work: a Systematic Review of the Research. INSEAD Working Paper No. 2015/48/OB, 2015. doi:10.2139/ssrn.2630650

10. Bachus V, Dishman L, Fick JW. Improving workforce experiences at United States Federally Qualified Health Centers: exploring the perceived impact of generational diversity on employee engagement. *Patient Exp J* 2022;**9**:17-30. doi:10.35680/2372-0247.1715

11. Hapsari C, Stoffers J, Gunawan A. The influence of generational diversity management and leader–member exchange on innovative work behaviors mediated by employee engagement. *J Asia Pac Bus* 2019;**20**:125-39. doi:10.1080/10599231.2019.1610684

12. World Bank. GDP growth (annual %). World Bank. Available: https://data.worldbank.org/indicator/NY.GDP.MKTP.KD.ZG [Accessed 13 Feb 2024].

13. Yi X, Ribbens B, Morgan CN. Generational differences in China: career implications. *Career Dev Int* 2010;**15**:601-20. doi:10.1108/13620431011084439

14. Cennamo L, Gardner D. Generational differences in work values, outcomes and person-organisation values fit. *J Manag Psychol* 2008;**23**:891-906. doi:10.1108/02683940810904385

15. Shragay D, Tziner A. The generational effect on the relationship between job involvement, work satisfaction, and organizational citizenship behavior. *J Work Organ Psychol* 2011;**27**:143-57. doi:10.5093/tr2011v27n2a6

16. Wang H, van Wijngaarden J, Buljac-Samardzic M, *et al*. Factors and interventions determining the functioning of health care teams in county-level hospitals in less affluent areas of China: a qualitative study. *Front Public Health* 2023;**11**:1082070. doi:10.3389/fpubh.2023.1082070

17. National Bureau of Statistics of China. Statistical communique of the People's Republic of China on the 2022 national economic and social development. National Bureau of Statistics of China; 2023. Available: http://www.stats.gov.cn/english/PressRelease/202302/t20230227_1918979.html [Accessed 13 Feb 2024].

18. Van de Klundert J, van Dongen-van den Broek J, Yesuf EM, *et al*. 'We are planning to leave, all of us'—a realist study of mechanisms explaining healthcare employee turnover in rural Ethiopia. *Hum Resour Health* 2018;**16**:37. doi:10.1186/s12960-018-0301-0

19. Van Emmerik IH, Brenninkmeijer V. Deep-level similarity and group social capital: associations with team functioning. *Small Group Res* 2009;**40**:650-69. doi:10.1177/1046496409346550

20. Williams HM, Parker SK, Turner N. Perceived dissimilarity and perspective taking within work teams. *Group Organ Manag* 2007;**32**:569-97. doi:10.1177/1059601106293769

21. Becker KL, Richards MB, Stollings J. Better together? Examining benefits and tensions of generational diversity and team performance. *J Int Relatsh*, 2022;**20**:442-63. doi:10.1080/15350770.2020.1837708

22. Tolbize A. *Generational differences in the workplace*. Minneapolis, MN: Research and Training Center on Community Living, 2008.

23. Jansen AE, Searle BJ. Diverse effects of team diversity: a review and framework of surface and deep-level diversity. *Pers Rev* 2021;**50**:1838-53. doi:10.1108/PR-12-2019-0664



For peer review only - http://bmjopen.bmj.com/site/about/guidelines.xhtml

24. Shemla M, Meyer B, Greer L, *et al*. A review of perceived diversity in teams: does how members perceive their team's composition affect team processes and outcomes?. *J Organ Behav* 2016;37:S89-106. doi:10.1002/job.1957

25. Byrne DE. *The attraction paradigm (Ser. Personality and psychopathology, 11)*. New York: Academic Press, 1971.

26. Mehra A, Kilduff M, Brass DJ. At the margins: a distinctiveness approach to the social identity and social networks of underrepresented groups. *Acad Manag J* 1998;**41**:441-52. doi:10.5465/257083

27. Tajfel H. Social identity and intergroup behaviour. *Soc Sci Inf* 1974;**13**:65-93. doi:10.1177/053901847401300204

28. Turner JC. Social categorization and the self-concept: a social cognitive theory of group behavior. In: Postmes T, Branscombe NR, editors. *Rediscovering Social Identity*. Psychology Press, 2010:243-72.

29. Kolbe M, Burtscher MJ, Wacker J, *et al*. Speaking up is related to better team performance in simulated anesthesia inductions: an observational study. *Anesth Analg* 2012;**115**:1099-108. doi:10.1213/ANE.0b013e318269cd32

30. Okuyama A, Wagner C, Bijnen B. Speaking up for patient safety by hospital-based health care professionals: a literature review. *BMC Health Serv Res* 2014;**14**:61. doi:10.1186/1472-6963-14-61

31. Henriksen K, Dayton E. Organizational silence and hidden threats to patient safety. *Health Serv Res* 2006;**41**:1539-54. doi:10.1111/j.1475-6773.2006.00564.x

32. Chang CW, Huang HC, Chiang CY, *et al*. Social capital and knowledge sharing: effects on patient safety. *J Adv Nurs* 2012;**68**:1793-803. doi:10.1111/j.1365-2648.2011.05871.x

33. Karamitri I, Talias MA, Bellali T. Knowledge management practices in healthcare settings: a systematic review. *Int J Health Plan Manag* 2017;**32**:4-18. doi:10.1002/hpm.2303

34. Kessel M, Kratzer J, Schultz C. Psychological safety, knowledge sharing, and creative performance in healthcare teams. *Creat Innov Manag* 2012;**21**:147-57. doi:10.1111/j.1467-8691.2012.00635.x

35. Moore S, Grunberg L, Krause AJ. Generational differences in workplace expectations: a comparison of production and professional workers. *Curr Psychol* 2015;**34**:346-62. doi:10.1007/s12144-014-9261-2

36. Liang J, Xu Y. Second-hand clothing consumption: a generational cohort analysis of the Chinese market. *Int J Consum Stud* 2018;**42**:120-30. doi:10.1111/ijcs.12393

37. Yi X, Ribbens B, Fu L, *et al*. Variation in career and workplace attitudes by generation, gender, and culture differences in career perceptions in the United States and China. *Employee Relat* 2015;**37**:66-82. doi:10.1108/ER-01-2014-0005

38. Zuo B, Lai Z. The effect of housing wealth on tourism consumption in China: age and generation cohort comparisons. *Tour Econ* 2020;**26**:211-32. doi:10.1177/1354816619861843

39. Morry MM, Kito MIE, Ortiz L. The attraction–similarity model and dating couples: projection, perceived similarity, and psychological benefits. *Pers Relatsh* 2011;**18**:125-43. doi:10.1111/j.1475-6811.2010.01293.x

40. Jones JS, Murray SR, Tapp SR. Generational differences in the workplace. *J Bus Divers* 2018;**18**:88-97. doi:10.33423/jbd.v18i2.528



For peer review only - http://bmjopen.bmj.com/site/about/guidelines.xhtml

41. Mowbray PK, Wilkinson A, Tse HH. An integrative review of employee voice: identifying a common conceptualization and research agenda. *Int J Manag Rev* 2015;**17**:382-400. doi:10.1111/ijmr.12045

42. Premeaux SF, Bedeian AG. Breaking the silence: the moderating effects of self-monitoring in predicting speaking up in the workplace. *J Manag Stud* 2003;**40**:1537-62. doi:10.1111/1467-6486.00390

43. Van Dyne L, Ang S, Botero IC. Conceptualizing employee silence and employee voice as multidimensional constructs. *J Manag Stud* 2003;**40**:1359-92. doi:10.1111/1467-6486.00384

44. Sherf EN, Parke MR, Isaakyan S. Distinguishing voice and silence at work: unique relationships with perceived impact, psychological safety, and burnout. *Acad Manag J* 2021;**64**:114-48. doi:10.5465/amj.2018.1428

45. Knoll M, Redman T. Does the presence of voice imply the absence of silence? The necessity to consider employees' affective attachment and job engagement. *Hum Resour Manag* 2016;**55**:829-44. doi:10.1002/hrm.21744

46. Qazi W, Raza SA, Khan KA. The contradiction between self-protection and self-presentation on knowledge sharing behaviour: evidence from higher education students in Pakistan. *Int J Knowl Learn* 2020;**13**:246-71. doi:10.1504/IJKL.2020.109910

47. Yi J. A measure of knowledge sharing behavior: scale development and validation. *Knowledge Manag Res Pract* 2009;**7**:65-81. doi:10.1057/kmrp.2008.36

48. Lainidi O, Jendeby MK, Montgomery A, *et al*. An integrative systematic review of employee silence and voice in healthcare: what are we really measuring?. *Front Psychiatry* 2023;**14**:1111579. doi:10.3389/fpsyt.2023.1111579

49. Lee SE, Choi J, Lee H, *et al*. Factors influencing nurses' willingness to speak up regarding patient safety in East Asia: a systematic review. *Risk Manag Healthc Policy* 2021;**14**:1053-63. doi:10.2147/RMHP.S297349

50. Lu J, Xie X. Research on employee silence behavior: a Review based on Chinese family enterprise. *Asian Soc Sci* 2013;**9**:47-52. doi:10.5539/ass.v9n17p47

51. Wang S, Noe RA. Knowledge sharing: a review and directions for future research. *Hum Resour Manag Rev* 2010;**20**:115-31. doi:10.1016/j.hrmr.2009.10.001

52. Deniz S, Çimen M. The mediating role of work alienation in the effect of workplace ostracism on employee voice. *Hospital Topics* 2022. doi:10.1080/00185868.2022.2116375

53. Imran MK, Fatima T, Sarwar A, *et al*. Will I speak up or remain silent? Workplace ostracism and employee performance based on self-control perspective. *J Soc Psychol* 2023;**163**:107-25. doi:10.1080/00224545.2021.1967843

54. Takhsha M, Barahimi N, Adelpanah A, *et al*. The effect of workplace ostracism on knowledge sharing: the mediating role of organization-based self-esteem and organizational silence. *J Workplace Learn* 2020;**32**:417-35. doi:10.1108/JWL-07-2019-0088



For peer review only - http://bmjopen.bmj.com/site/about/guidelines.xhtml

55. Wang GH, Li JH, Liu H, *et al*. The association between workplace ostracism and knowledge-sharing behaviors among Chinese university teachers: the chain mediating model of job burnout and job satisfaction. *Front Psychol* 2023;14:1030043. doi:10.3389/fpsyg.2023.1030043

56. Gkorezis P, Panagiotou M, Theodorou M. Workplace ostracism and employee silence in nursing: the mediating role of organizational identification. *J Adv Nurs* 2016;**72**:2381-88. doi:10.1111/jan.12992

57. Bhatti SH, Hussain M, Santoro G, *et al*. The impact of organizational ostracism on knowledge hiding: analysing the sequential mediating role of efficacy needs and psychological distress. *J Knowl Manag* 2023;**27**:485-505. doi:10.1108/JKM-03-2021-0223

58. Zhao H, Xia Q, He P, *et al*. Workplace ostracism and knowledge hiding in service organizations. *Int J Hosp Manag* 2016;**59**:84-94. doi:10.1016/j.ijhm.2016.09.009

59. Blau PM. *Inequality and heterogeneity: A primitive theory of social structure*. New York: Free Press, 1977.

60. Liden RC, Wayne SJ, Stilwell D. A longitudinal study on the early development of leader-member exchanges. *J Appl Psychol* 1993;**78**:662-74. doi: 10.1037/0021-9010.78.4.662

61. Van Dyne L, LePine JA. Helping and voice extra-role behaviors: evidence of construct and predictive validity. *Acad Manag J* 1998;**41**:108-19. doi:10.5465/256902

62. Morrison EW, Wheeler-Smith SL, Kamdar D. Speaking up in groups: a cross-level study of group voice climate and voice. *J Appl Psychol* 2011;**96**:183-91. doi:10.1037/a0020744

63. Detert JR, Edmondson AC. Implicit voice theories: taken-for-granted rules of self-censorship at work. *Acad Manag J* 2022;**54**:461-88. doi:10.5465/amj.2011.61967925

64. Guenter H, Schreurs B, van Emmerik IH, *et al*. What does it take to break the silence in teams: authentic leadership and/or proactive followership?. *Appl Psychol* 2017;**66**:49-77. doi:10.1111/apps.12076

65. Mignonac K, Herrbach O, Serrano Archimi C, *et al*. Navigating ambivalence: perceived organizational prestige–support discrepancy and its relation to employee cynicism and silence. *J Manag Stud* 2018;**55**:837-72. doi:10.1111/joms.12330

66. Pittino D, Martínez AB, Chirico F, *et al*. Psychological ownership, knowledge sharing and entrepreneurial orientation in family firms: the moderating role of governance heterogeneity. *J Bus Res* 2018;**84**:312-26. doi:10.1016/j.jbusres.2017.08.014

67. Bartol KM, Liu W, Zeng X, *et al*. Social exchange and knowledge sharing among knowledge workers: the moderating role of perceived job security. *Manag Organ Rev* 2009;**5**:223-40. doi:10.1111/j.1740-8784.2009.00146.x

68. Dong Y, Bartol KM, Zhang ZX, *et al*. Enhancing employee creativity via individual skill development and team knowledge sharing: Influences of dual-focused transformational leadership. *J Organ Behav* 2017;**38**:439-58. doi:10.1002/job.2134

69. LePine JA, Van Dyne L. Predicting voice behavior in work groups. *J Appl Psychol* 1998;**83**:853-68. doi:10.1037/0021-9010.83.6.853



For peer review only - http://bmjopen.bmj.com/site/about/guidelines.xhtml

70. Post C. When is female leadership an advantage? Coordination requirements, team cohesion, and team interaction norms. *J Organ Behav* 2015;**36**:1153-75. doi:10.1002/job.2031

71. Behling O, Law KS. *Translating questionnaires and other research instruments: problems and solutions*. Thousand Oaks, CA: Sage, 2000. doi:10.4135/9781412986373

72. Bell A, Fairbrother M, Jones K. Fixed and random effects models: making an informed choice. *Qual Quant* 2019;**53**:1051-74. doi:10.1007/s11135-018-0802-x

73. Rockwood NJ. *Advancing the formulation and testing of multilevel mediation and moderated mediation models (Master's thesis)*. The Ohio State University, Columbus, OH, 2017.

74. Von Elm E, Altman DG, Egger M, *et al*. The Strengthening the Reporting of Observational Studies in Epidemiology (STROBE) statement: guidelines for reporting observational studies. *Lancet* 2007;**370**:1453-57. doi:10.7326/0003-4819-147-8-200710160-00

75. National Health Commission. The statistical bulletin of the development of health services in China in 2022. National Health Commission; 2023. Available: http://www.nhc.gov.cn/cms-search/downFiles/9b3fddc4703d4c9d9ad399bcca089f03.pdf [Accessed 13 Feb 2024].

76. Kim JH. Multicollinearity and misleading statistical results. *Korean J Anesthesiol* 2019;**72**:558-69. doi:10.4097/kja.19087

77. Jehn KA, Northcraft GB, Neale MA. Why differences make a difference: A field study of diversity, conflict and performance in workgroups. *Adm Sci Q* 1999;**44**:741-63. doi:10.2307/2667054

78. Ott-Holland CJ, Huang JL, Ryan AM, *et al*. The effects of culture and gender on perceived self-other similarity in personality. *J Res Personal* 2014;**53**:13-21. doi:10.1016/j.jrp.2014.07.010

79. Cardon PW, Scott JC. Chinese business face: communication behaviors and teaching approaches. *Bus Commun Q* 2003;**66**:9-22. doi:10.1177/108056990306600402

80. Becker JC, Tausch N. When group memberships are negative: the concept, measurement, and behavioral implications of psychological disidentification. *Self Identity* 2014;**13**:294-321. doi:10.1080/15298868.2013.819991

81. Sultana MS, Niu X, Hossain MS. How dissimilarity attributes at restaurants trigger negative emotions and associated behavioral intentions: the role of attribute performance. *J Hosp and Tour Insights* 2023;6:2199-221. doi:10.1108/JHTI-03-2022-0115



For peer review only - http://bmjopen.bmj.com/site/about/guidelines.xhtml

## Appendix 1. Measures for perceived similarity, speaking up, silence and knowledge sharing

### *Perceived similarity*[20,60]

1. Team members handle problems in a similar way.

2. Team members think alike in terms of coming up with a similar solution.

3. Team members analyse problems in a similar way.

4. Team members see things in much the same way.

5. Team members are similar in terms of outlook and values.

6. Team members are alike in a number of areas.

### *Speaking up*[61,62]

1. I develop and make recommendations concerning issues that affect this team.

2. I speak up and encourage others in this team to get involved in issues that affect this team.

3. I communicate my opinions about work issues to others in this team even if my opinion is different and

4. I keep well informed about issues where my opinion might be useful to this team.

5. I get involved in issues that affect the quality of work life here in this team.

6. I speak up in this team with ideas for new projects or changes in procedures.

### *Silence*[63-65]

1. I withhold ideas from the team leader for changing inefficient work policies.

2. I keep ideas for developing new products or services to myself.

3. I do not speak up about difficulties caused by the way the team leader and the team members interact.

4. I keep quiet in team meetings about problems with daily routines that hamper performance.

5. I withhold thoughts about improving patients' experiences with us.

### *Knowledge sharing*[66,67]

1. Team members share information that can be helpful to the team.

2. Team members keep information flow high to increase team effectiveness.

3. Team members seek helpful information to be shared within the team.

For peer review only

4. Team members share expertise to help resolve the team's problems.

5. Team members collectively offer innovative ideas that can benefit the team.

For peer review only

**Appendix 2.** *Additional multilevel mediation analysis (Diversity in the composition of local and non-local healthcare professionals)*

| | Perceived similarity | Speak up | Silence | Knowledge sharing |
|---|---|---|---|---|
| ***Fixed effects*** | | | | |
| **Within-group (Level 1)** | | | | |
| Intercept | 5.54** | 1.76** | 1.62** | 2.39** |
| **Perceived similarity** | - | 0.56** | 0.39** | 0.54** |
| Gender | 0.23 | 0.04 | -0.31 | 0.30** |
| Team tenure | -0.00 | 0.00 | 0.01 | -0.01 |
| **Between-group (Level 2)** | | | | |
| **Diversity in composition** | -0.22 | 0.08 | 0.50 | 0.16 |
| **Perceived similarity** | - | 0.70** | 0.40** | 0.60** |
| Gender | 0.25 | -0.14 | 0.15 | 0.16 |
| Team tenure | 0.01 | 0.01 | 0.03 | 0.00 |
| Team size | -0.00 | -0.00 | -0.00 | -0.00 |
| **Indirect effect (Mediation)** | - | -0.16 | -0.09 | -0.14 |
| ***Random effects (Variance)*** | | | | |
| Intercept | 0.19** | 0.03 | 0.40** | 0.04 |
| Slope | - | 0.12** | 0.04 | 0.11** |

**: p<0.05; **: p<0.01*

For peer review only

BMJ Open

# Does (generational) diversity improve team functioning in rural Chinese hospitals? A cross-sectional survey study



| | |
|---|---|
| Journal: | *BMJ Open* |
| Manuscript ID | bmjopen-2024-086451.R1 |
| Article Type: | Original research |
| Date Submitted by the Author: | 25-Sep-2024 |
| Complete List of Authors: | Wang, Hujie; Erasmus University Rotterdam, Erasmus School of Health Policy & Management<br>Buljac-Samardžić, Martina; Erasmus University Rotterdam, Erasmus School of Health Policy & Management<br>Van Wijngaarden, Jeroen; Erasmus Universiteit Rotterdam, Erasmus School of Health Policy & Management<br>van de Klundert, Joris; Erasmus University Rotterdam, Erasmus School of Health Policy & Management; Universidad Adolfo Ibáñez |
| <b>Primary Subject Heading</b>: | Health services research |
| Secondary Subject Heading: | Health policy, Health services research |
| Keywords: | Health Services, Human resource management < HEALTH SERVICES ADMINISTRATION & MANAGEMENT, Health policy < HEALTH SERVICES ADMINISTRATION & MANAGEMENT, Health Workforce |
| | |



SCHOLARONE™
Manuscripts

*I, the Submitting Author has the right to grant and does grant on behalf of all authors of the Work (as defined in the below author licence), an exclusive licence and/or a non-exclusive licence for contributions from authors who are: i) UK Crown employees; ii) where BMJ has agreed a CC-BY licence shall apply, and/or iii) in accordance with the terms applicable for US Federal Government officers or employees acting as part of their official duties; on a worldwide, perpetual, irrevocable, royalty-free basis to BMJ Publishing Group Ltd ("BMJ") its licensees and where the relevant Journal is co-owned by BMJ to the co-owners of the Journal, to publish the Work in this journal and any other BMJ products and to exploit all rights, as set out in our licence.*

*The Submitting Author accepts and understands that any supply made under these terms is made by BMJ to the Submitting Author unless you are acting as an employee on behalf of your employer or a postgraduate student of an affiliated institution which is paying any applicable article publishing charge ("APC") for Open Access articles. Where the Submitting Author wishes to make the Work available on an Open Access basis (and intends to pay the relevant APC), the terms of reuse of such Open Access shall be governed by a Creative Commons licence – details of these licences and which Creative Commons licence will apply to this Work are set out in our licence referred to above.*

*Other than as permitted in any relevant BMJ Author's Self Archiving Policies, I confirm this Work has not been accepted for publication elsewhere, is not being considered for publication elsewhere and does not duplicate material already published. I confirm all authors consent to publication of this Work and authorise the granting of this licence.*

For peer review only

# Does (generational) diversity improve team functioning in rural Chinese hospitals? A cross-sectional survey study

**Hujie Wang[1*], Martina Buljac-Samardzic[1], Jeroen van Wijngaarden[1], Joris van de Klundert[1,2]**

[1] Erasmus School of Health Policy & Management, Erasmus University Rotterdam, Rotterdam, the Netherlands
[2] School of Business, Universidad Adolfo Ibáñez, Santiago, Chile
* **Correspondence**: Hujie Wang (wang@eshpm.eur.nl)

## Abstract

**Objectives**: Generational diversity, increasingly prominent in the composition of the healthcare workforce in rapidly developing countries, has received much attention in practice and research recently. While research has revealed various positive and negative impacts of generational diversity on team functioning, the understanding of the mechanism explaining how generational diversity influences team functioning is still limited. This study in rural Chinese hospitals examines the relationship between (surface-level) generational diversity and (deep-level) perceived similarity and investigates how they influence three teamwork behaviours that importantly determine quality of care, namely, speaking up, silence and knowledge sharing.

**Design**: We adopted a quantitative research design and conducted an online survey to investigate the relationship among generational diversity, perceived similarity, speaking up, silence and knowledge sharing. Multilevel mediation modelling was used to test the hypotheses.

Setting: The study was conducted in four rural Chinese hospitals.

Participants: 841 healthcare professionals, including doctors, nurses and other healthcare professionals, were included in the study.

Primary and secondary outcome measures: Generational diversity was measured by calculating the average of individuals' Blau's Indices regarding all the generations for each team. Perceived similarity, speaking up, silence and knowledge sharing were measured by validated questionnaires from literature.

**Results**: Perceived similarity is positively related to the three teamwork behaviours, i.e. speaking up ($\beta=0.56$, $p<0.01$), silence ($\beta=0.39$, $p<0.01$) and knowledge sharing ($\beta=0.54$, $p<0.01$), while generational diversity is not (speaking up: $\beta=0.08$, $p>0.05$; silence: $\beta=0.44$, $p>0.05$; knowledge sharing: $\beta=0.09$, $p>0.05$). As the relationship between generational diversity and perceived similarity is non-significant ($\beta=0.07$, $p>0.05$), perceived similarity does not mediate the relationship between generational diversity and teamwork behaviour.

**Conclusion**: The findings suggest that increases in generational diversity that results from healthcare workforce strengthening may not impact team behaviours and performance. However, if healthcare workforce strengthening would reduce the perceived similarity in teams, explicit management efforts to mitigate the negative impact on team behaviour and care provision are called for.



For peer review only - http://bmjopen.bmj.com/site/about/guidelines.xhtml

***Keywords***: generational diversity, perceived similarity, teamwork, speaking up, silence, knowledge sharing

**Strengths and limitations of this study**

- Multilevel analysis was used to provide more accurate insights into the relationships among the variables by eliminating the cluster effects of the data.
- Cross-sectional design does not allow to claim causal relationships.
- Using one method to collect data from the same respondents may produce common method bias and common resource bias.

**Introduction**

Sustainable Development Goal 3 (SDG3) aims at good health and well-being for all and at universal health coverage (UHC).[1] However, many developing countries experience barriers to high-quality care delivery, a key component of UHC, due to healthcare workforce shortages. These shortages are particularly severe in rural and remote areas.[2-4] Improving healthcare workforce management in developing countries, including recruitment, development and retention, can be an effective strategy towards achieving UHC.[1] Attracting and retaining a highly qualified workforce often requires embracing diversity in the workforce and healthcare teams. This diversity includes variation in generations, professional background and cultural or geographical origins.

Workforce diversity can both positively (e.g. better health outcomes) and negatively (e.g. increased conflict) impact healthcare team performance.[5,6] However, evidence is predominantly from developed countries,[5,6] leaving the role of workforce diversity in the performance of rural healthcare teams in developing countries, where UHC is most challenging, under-researched.

This study focuses on diversity of healthcare teams, particularly generational diversity, as a potential pathway to address workforce shortages and provide high quality care.[7] The evidence regarding the relationship between generational diversity and team functioning is limited, inconclusive and primarily from developed countries.[8,9] For example, generational diversity may promote innovative team behaviour but also create communication barriers.[10,11] We aim to expand the evidence base by examining the relationship between diversity, particularly generational diversity, and healthcare team functioning in rural Chinese hospitals.

In recent decades, many developing countries such as China have experienced rapid and profound economic development and social changes, leading to larger generational differences compared to developed countries with more modest development rates. For instance, since initiating social reforms and opening-up in 1978, China has achieved an average yearly GDP growth rate of 9.1%. This rate is nearly threefold the 3.1% yearly growth of the global GDP during the same period.[12] Such growth can amplify the differences in behaviours and values between generations;[13] a generation is defined as a group of people sharing birth years in a certain period and raised in a similar social and development stage.[14,15] These differences in behaviours and values may subsequently influence team functioning and performance.

Rural Chinese hospitals have traditionally employed a less diverse workforce, mostly drawn from the local population, and have struggled with attracting highly educated healthcare professionals.[16] For these rural hospitals, which serve nearly half a billion rural Chinese citizens,[17] recruiting young,



For peer review only - http://bmjopen.bmj.com/site/about/guidelines.xhtml

highly educated and nonlocal professionals increases (generational) diversity and supports delivering high-quality care as part of UHC.[1] To the best of our knowledge, existing evidence on the challenges posed by (generational) diversity in rural healthcare teams, both in China and elsewhere, is scarce and predominantly qualitative.[2,16,18] This study presents a first quantitative study into the relationship between diversity and teamwork behaviour in rural Chinese hospital teams.

Generational diversity is based on demographic characteristics and is therefore considered a form of surface-level diversity.[19,20] It may lead to perceived (dis)similarities between generations regarding communication styles and work attitudes.[21,22] These perceived (dis)similarities are forms of deep-level diversity, as they involve underlying attributes such as beliefs, attitudes and values.[20] Jansen and Searle's review on team diversity recommends researching surface-level and deep-level diversity simultaneously.[23]

Empirical evidence and established theories support a relationship between perceived similarity and team functioning. Shemla et al.'s review reports that perceived similarity is positively related to team members' supportive behaviour (i.e. social and task exchange) and team commitment while reducing employees' intention to resign.[24] Jansen and Searle's review also highlights the benefit of perceived similarity for team performance, team efficiency and job satisfaction.[23] The similarity attraction theory[25] suggests that similar team members are more likely to understand each other's thoughts and behaviours, increasing interaction.[20,26] In addition, social identity theory[27] and self-categorisation theory[28] propose that team members may categorise similar team members as "ingroups" with whom they prefer to interact, while less similar team members are viewed as "outgroups", resulting in less interaction.

As perceived similarity may be directly associated with communication and interaction, we operationalise team functioning through three individual behaviours essential to healthcare delivery: speaking up, silence and knowledge sharing. Silence and speaking up refer to the extent individuals voice work-related issues. Research has demonstrated the positive effect of speaking up[29,30] and the negative effect of silence[31] on team functioning in healthcare. Over the past years, knowledge sharing has also gained attention in healthcare.[32-34] Team members might still share knowledge to promote team functioning even when the team climate does not support speaking up or when it is not seen as appropriate.

If generational diversity translates into perceived similarity, which in turn impacts speaking up, silence and knowledge sharing, perceived similarity acts as a mediating mechanism through which generational diversity impacts the three teamwork behaviours. This study investigates how generational diversity and perceived similarity influence speaking up, silence and knowledge sharing in rural Chinese hospitals.

## Hypotheses

### *Relationship between generational diversity and perceived similarity*

Generational diversity occurs when a team includes members from at least two generations. Commonly considered generations in scientific literature include Baby Boomer (born between 1946 and 1964), Generation X (born between 1965 and 1981) and Generation Y (born between 1982 and 2000).[35] This categorisation is rooted in Western context and evidence, and it may not have external validity in rural China which has developed quite differently. In this study, we follow the generational categorisation commonly used in China-related literature which distinguishes the decades "1960s,



For peer review only - http://bmjopen.bmj.com/site/about/guidelines.xhtml

1970s, …" to define corresponding generations.[13,36-38] Each of these generations possesses distinct characteristics that are relevant to team functioning. For example, in China, people born in the 1970s are more collectivist and pragmatic, while those born in the 1980s are more individualistic and confident.[13,37]

Perceived similarity refers to one's perception of the similarity between people[39] and is related to deep-level attributes (i.e. underlying elements such as values, attitudes and beliefs).[20] (Surface-level) generational diversity may underpin perceived similarity through generational differences in values, attitudes and beliefs.[8,40] In a generationally diverse team, people from different generations may hold distinct values, attitudes and beliefs and will, therefore, perceive those from other generations as dissimilar to themselves. For the same reason, team members may be more likely to consider other team members from the same generation to be as similar to themselves. Therefore, we propose:

**Hypothesis 1**: Generational diversity is negatively related to perceived similarity.

***Relationship between perceived similarity and teamwork behaviours***

Speaking up, also known as voice behaviour, is defined as expressing one's opinions about any work-related matters.[41,42] Employee silence refers to "withholding ideas, information and opinions" relevant to the improvements in workplace.[43] Speaking up and silence are not necessarily opposites and can coexist. Individuals who speak up on certain issues may still intentionally withhold their ideas and keep silent about other issues at the same time.[43,44] Knoll and Redman even found that some types of speaking up and silence behaviours (i.e. promotive speaking up and cooperative silence) are positively correlated.[45]

Sometimes, team members may not be able or willing to point out work-related issues but may still practice knowledge sharing. Knowledge sharing refers to people sharing their work-related knowledge with team members to help improve team effectiveness.[46,47]

Antecedents of speaking up, silence and knowledge sharing are identified by previous reviews and studies. For example, some reviews summarise the factors influencing speaking up and silence behaviour (e.g., motivation, contextual factors, individual factors and organisational factors).[30,48-50] S. Wang & Noe have studied the factors influencing knowledge sharing and provide evidence that diversity in demographic features (e.g., gender, education and marital status) is associated with knowledge sharing in non-healthcare settings.[51] Workplace ostracism has been shown to be negatively related to speaking up[52,53] and knowledge sharing[54.55] but to be positively associated with silence[53,56] and knowledge hiding.[57,58]

The similarity attraction theory suggests that people are attracted to those similar to themselves but may ostracise those who are dissimilar.[25] According to this theory, individuals more easily build good interpersonal relations with similar ones, understand their thoughts and behaviours better and are subsequently more likely to interact with them.[20,26] In line with this reasoning, team members would be more likely to speak up and share knowledge within the team and less likely to withhold their ideas if they perceive themselves to be more similar to each other. Conversely, team members would be more reluctant to express their ideas and share their knowledge and more prone to keeping silent with team members perceived as dissimilar. Therefore, we propose:

**Hypothesis 2a**: Perceived similarity is positively related to speaking up.



For peer review only - http://bmjopen.bmj.com/site/about/guidelines.xhtml

**Hypothesis 2b**: Perceived similarity is negatively related to silence.

**Hypothesis 2c**: Perceived similarity is positively related to knowledge sharing.

### *The mediating role of perceived similarity*

The similarity attraction theory posits that individuals' actual similarity (e.g., demographic characteristics) leads to perceived similarity and consequently influence behaviour.[25] As generational diversity creates dissimilarity, individuals may perceive team members from other generations as dissimilar, causing them to be less likely to speak up, more likely to remain silent and less likely to share knowledge. Similarly, low generational diversity within teams, as occurs when there are few generational differences between team members, would imply high perceived similarity, promoting speaking up and knowledge sharing and reducing silence. We accordingly propose:

**Hypothesis 3**: Perceived similarity mediates the relationship between generational diversity and a) speaking up, b) silence and c) knowledge sharing.

The theoretical model of this study is shown in **Figure 1**.

*Figure 1. Theoretical Model*

## Methods

### *Sample and procedure*

Following our research aims, we approached rural hospitals in China. Specifically, we approached seven county-level hospitals, with the help of the Health Human Resources Development Centre of the National Health Commission of China and the County Health Media. The former is responsible for promoting the human resource management for hospitals of all levels in China, while the latter is a media company focusing on China's county-level hospitals. Four out of the seven county-level hospitals accepted our invitation to participate in the study. These four hospitals are located in four different provincial administrative regions across China. Together, these four hospitals employ 3500 employees, including approximately 1000 doctors and 1700 nurses.

This study was conducted anonymously through an online cross-sectional survey, which was disseminated via the widely used Chinese survey platform "Wen Juan Xing" in October 2022. The hospital presidents of the four participating hospitals explained the research aims to the team leaders and asked them to share the survey link with team members. Participants were team members, including doctors, nurses and other healthcare professionals, working in monodisciplinary teams at the four county-level hospitals. Team leaders were included only for the measurement of generational diversity, but not for the other constructs. Each respondent was restricted by the survey platform to submit only one response per item.

The Research Ethics Review Committee of Erasmus School of Health Policy and Management, Erasmus University Rotterdam approved this study (No. ETH2122-0807). Consent was obtained from each respondent before the data collection.



For peer review only - http://bmjopen.bmj.com/site/about/guidelines.xhtml

### Measures

***Generational diversity***. Following existing literature on the definition of generations in China, we specified the generations into five categories: born between 1960 and 1969, born between 1970 and 1979, born between 1980 and 1989, born between 1990 and 1999, and born after 2000.[13,36-38] Team members and team leaders were asked to report the number of people for each category on their teams. The generational diversity perceived by each participant was calculated using Blau's index[59]: $1-\sum_{i=1}^{n} p_i^2$, where $p_i$ is the perceived proportion of team members in the $i^{th}$ category (generation), and $n$ is the perceived number of categories (generations) in the team. Blau's index equals 0 if all team members are from the same generation and increases as the members are divided across more generations. As generational diversity is a team-level construct, we calculated the mean of the individual Blau's indices of team members and team leaders for each team to express perceived generational diversity at the team-level.

***Perceived similarity***. We measured the perceived similarity between team members using Williams et al.'s six-item measure,[20] which was adapted from Liden et al.[60] This scale is a seven-point Likert scale, ranging from 1 (strongly disagree) to 7 (strongly agree). The Cronbach's α in this study was 0.97.

***Speaking up***. Speaking up was measured by a six-item measure, developed by van Dyne and LePine[61] and adapted by Morrison et al.[62] This seven-point Likert scale ranged from 1 (strongly disagree) to 7 (strongly agree). The Cronbach's α in this study was 0.94.

***Silence***. A five-item measure developed by Detert and Edmonson[63] and adapted by Guenter et al.[64] and Mignonac et al.[65] was used to test employee silence. The items were rated on a seven-point Likert scale ranging from 1 (strongly disagree) to 7 (strongly agree). The Cronbach's α in this study was 0.94**.**

***Knowledge sharing***. We assessed knowledge sharing with Pittino et al.'s five-item measure,[66] as adapted from Bartol et al.[67] A seven-point Likert scale was used, ranging from 1 (strongly disagree) to 7 (strongly agree). The Cronbach's α in this study was 0.99.

***Control variables***. From a wide set of literature based control variables, gender and team tenure were significantly associated with one of the dependent variables in preliminary analyses and were therefore included.[20,61,62,64,67,68] Furthermore, we also controlled for team size at the team level as it has been found to influence speaking up and other interaction related variables within a team in previous research.[69,70]

The measures for perceived similarity, speaking up, silence and knowledge sharing are presented in Appendix 1. All measures were translated from English to Chinese using the standard translation/back-translation technique.[71] The average scores of the items for each scale were used to form the individual measurements of perceived similarity, speaking up, silence and knowledge sharing for each participant.

### Analysis

All the analyses were conducted via SPSS 29 and AMOS 28. We conducted descriptive and correlation analyses via SPSS and confirmatory factor analysis (CFA) via AMOS to provide an overview of the data. The CFA shows good factor loadings for perceived similarity, speaking up, silence and knowledge sharing, ranging from 0.72 to 0.99. The model fit indices of the CFA suggest



For peer review only - http://bmjopen.bmj.com/site/about/guidelines.xhtml

that the four-factor model (i.e., a model with perceived similarity, speaking up, silence and knowledge sharing being separate factors) presents an acceptable model fit ($\chi^2(203)=1796.92$, CFI=0.94, TLI=0.94, RMSEA=0.10, SRMR=0.05) and fits significantly better than the three-factor model (i.e. combining speaking up and silence into one factor; $\chi^2(206)=5491.54$, $p<0.01$, CFI=0.81, TLI=0.79, RMSEA=0.18, SRMR=0.14) , two-factor model (i.e. combining speaking up, silence and knowledge sharing; $\chi^2(208)=8163.26$, $p<0.01$, CFI=0.71, TLI=0.68, RMSEA=0.21, SRMR=0.18) and one-factor model (i.e. combining all the four as one factor; $\chi^2(209)=12430.70$, $p<0.01$, CFI=0.56, TLI=0.51, RMSEA=0.26, SRMR=0.21).

Multilevel analysis was used to analyse the data as respondents were nested within teams, and teams were further nested within hospitals, which caused dependencies between observations. The standard mixed linear models in SPSS showed that there were significant between-team variances for the three dependent variables (i.e. speaking up, silence and knowledge sharing), indicating the necessity of conducting a multilevel analysis. In addition, between-hospital variances were not significant. Therefore, a two-level mediation analysis was performed; within-group (level 1) represented the individual level, while between-group (level 2) represented the team level. We chose the random effects model for the multilevel mediation analysis as it is expected that the intercepts and slopes would vary across higher-level groups (i.e. teams).[72]

We used the MLmed macro in SPSS to conduct the multilevel mediation analysis.[73] In the interface of the MLmed macro, three different random effects models were built with "speaking up", "silence" and "knowledge sharing" (level 1) as dependent variables. "Generational diversity" (level 2) served as the independent variable, while "perceived similarity" (level 1) served as a mediator. These three models were 2-1-1 multilevel mediation models in which the independent variable was a team-level (level 2) variable, and the other variables were at the individual level (level 1). The control variables gender and team tenure were level 1 covariates, and team size was a level 2 covariate.

As generational diversity is a level 2 variable, the random-effect parameters only account for the intercepts of the mediator and dependent variables and the slopes regarding the relationships between perceived similarity and the three behavioural variables (i.e., speaking up, silence and knowledge sharing) as these four variables are all at level 1.

Additional multilevel mediation analyses replacing generational diversity by the diversity in the composition of local and non-local healthcare professionals were conducted to provide more insights for future research. The reporting of this study follows the Checklist for Reporting of Survey Studies (CROSS).[74]

### *Patient and public involvement*

Patients or the public were not involved in the design and reporting of our research. Nevertheless, the Health Human Resources Development Centre of the National Health Commission of China and the hospital management of the four participating hospitals play a supporting role in conducting the research, aiming for quality and teamwork improvement in all rural hospitals. Therefore, we will disseminate our findings with the participating hospitals to improve team functioning and subsequently the quality of care.

## Results



For peer review only - http://bmjopen.bmj.com/site/about/guidelines.xhtml

### *Descriptive analysis*

A total of 841 valid questionnaires were received. The average age of the respondents is 32.10 years (median: 31.00; standard deviation: 8.08). The average team tenure is 6.71 years (median: 5.00; standard deviation: 6.37). The percentage of doctors among the respondents (38.64%) is similar to that in all Chinese hospitals (38.04%), while the percentage of nurses (56.96%) is higher than the national data (44.81%).[75] The respondents' demographic characteristics are shown in Table 1.

*Table 1. Demographic characteristics*

| | **Number of people (Percentage)** |
|---|---|
| **Gender** | |
| Male | 165 (19.62%) |
| Female | 652 (77.53%) |
| Prefer not to say | 24 (2.85%) |
| **Age*** | |
| <=30 | 414 (49.23%) |
| 31-40 | 287 (34.13%) |
| 41-50 | 103 (12.25%) |
| >=51 | 32 (3.80%) |
| **Profession** | |
| Doctors | 325 (38.64%) |
| Nurses | 479 (56.96%) |
| Other healthcare professionals | 37 (4.40%) |
| **Local or non-local** | |
| Local | 731 (86.92%) |
| Non-local | 110 (13.08%) |
| **Education background** | |
| Master | 18 (2.14%) |
| Bachelor | 593 (70.51%) |
| Lower than bachelor | 230 (27.35%) |
| **Professional title** | |
| Senior | 17 (2.02%) |
| Deputy senior | 55 (6.54%) |
| Intermediate | 215 (25.56%) |
| Junior | 554 (65.87%) |

** There are missing values regarding age, so the sum of the number of people per age group is smaller than the total number of respondents.*

The correlation analyses (Table 2) show significant strong correlations between perceived similarity on the one hand and speaking up ($r$= 0.65, $p$<0.01) and knowledge sharing ($r$= 0.67, $p$<0.01) on the other hand. The analysis finds a small to moderate correlation between perceived similarity and silence ($r$=0.26, $p$<0.01). Significant strong correlation is also shown between speaking up and knowledge sharing ($r$= 0.75, $p$<0.01), while the positive correlation between speaking up and silence is moderate ($r$=0.33, $p$<0.01). Generational diversity at team level is not significantly correlated to either perceived similarity or individual behaviours. The full collinearity test shows that the values of the variance inflation factor for all the control variables, independent variable and mediator range from 1.01 to 1.12, indicating no multicollinearity issues in this study.[76]



For peer review only

*Table 2. The correlation matrix of all variables*

| | 1 | 2 | 3 | 4 | 5 | 6 | 7 | 8 |
|---|---|---|---|---|---|---|---|---|
| 1. Gender (1=female) | 1.00 | | | | | | | |
| 2. Team tenure | -0.19** | 1.00 | | | | | | |
| 3. Team size | 0.02 | 0.10** | 1.00 | | | | | |
| 4. Generational diversity*** | -0.14** | 0.23** | 0.22** | 1.00 | | | | |
| 5. Perceived similarity | 0.07 | 0.00 | -0.03 | -0.00 | 1.00 | | | |
| 6. Speaking up | 0.02 | 0.04 | -0.06 | 0.01 | 0.65** | 1.00 | | |
| 7. Silence | -0.02 | 0.06 | -0.01 | 0.04 | 0.26** | 0.33** | 1.00 | |
| 8. Knowledge sharing | 0.12** | -0.01 | -0.03 | 0.00 | 0.67** | 0.75** | 0.15** | 1.00 |

***: p<0.01*

****: The generational diversity is a team-level construct, and thus a single value is assigned to all the individuals from the same team within the range of 0 and 0.8.*

***Multilevel mediation analysis***

The three 2-1-1 multilevel mediation models are shown in Table 3.

*Table 3. Multilevel mediation analyses*

| | **Perceived similarity** | **Speaking up** | **Silence** | **Knowledge sharing** |
|---|---|---|---|---|
| ***Fixed effects*** | | | | |
| **Within-group (Level 1)** | | | | |
| Intercept | 5.46** | 1.75** | 1.65* | 2.39** |
| **Perceived similarity** | - | 0.56** | 0.39** | 0.54** |
| Gender | 0.23 | 0.04 | -0.31 | 0.30** |
| Team tenure | -0.00 | 0.00 | 0.01 | -0.01 |
| **Between-group (Level 2)** | | | | |
| **Generational diversity** | 0.07 | 0.08 | 0.44 | 0.09 |
| **Perceived similarity** | - | 0.69** | 0.38** | 0.60** |
| Gender | 0.27 | -0.15 | 0.11 | 0.14 |
| Team tenure | 0.01 | 0.01 | 0.02 | 0.00 |
| Team size | -0.00 | -0.00 | -0.00 | -0.00 |
| **Indirect effect (Mediation)** | - | 0.05 | 0.03 | 0.04 |
| ***Random effects (Variance)*** | | | | |
| Intercept | 0.19** | 0.03 | 0.41** | 0.04 |
| Slope | - | 0.12** | 0.04 | 0.11** |

**: p<0.05; **: p<0.01*

Table 3 shows that there is no significant relationship between generational diversity and perceived similarity ($\beta$=-0.07, $p$>0.05). Therefore, hypothesis 1 is rejected, which also violates the first requirement for a mediating relationship.



For peer review only - http://bmjopen.bmj.com/site/about/guidelines.xhtml
For peer review only

The results also show that perceived similarity is positively related to all the three behavioural variables at both the within-group level (speaking up: β= 0.56, p<0.01; silence: β=0.39, p<0.01; knowledge sharing: β=0.54, p<0.01) and between-group level (speaking up: β=0.69, p<0.01; silence: β=0.38, p<0.01; knowledge sharing: β=0.60, p<0.01). These findings indicate that people will be more likely to speak up, keep silent and share knowledge when they perceive themselves to be more similar to team members. As a result, these results support hypotheses 2a and 2c but reject hypothesis 2b, which specifies a negative relationship between perceived similarity and silence.

The random-effect parameters show that the relationships between perceived similarity and speaking up (0.12, p<0.01) and between perceived similarity and knowledge sharing (0.11, p<0.01) significantly vary across groups, while the relationship between perceived similarity and silence (0.04, p>0.05) does not significantly change between groups. These findings illustrate that within the teams with the same extent of perceived similarity, team members in some of these teams are more willing to speak up and share knowledge than those in some other teams, but the extent of keeping silent does not vary across teams. Furthermore, the intercepts of perceived similarity and silence vary across groups, while those of speaking up and knowledge sharing do not. These results indicate that team members from different teams have different baseline degrees of the perceptions towards other team members and keeping silent. However, the baseline extents of speaking up and sharing knowledge remain constant across teams.

As generational diversity is a level 2 variable, only between-group direct effects (i.e., the relationships between generational diversity and the three behavioural variables) and mediated effects (i.e., indirect effects) are evaluated. As shown in Table 3, there are neither significant direct effects of generational diversity on the three behavioural variables (speaking up: β=0.08, p>0.05; silence: β=0.44, p>0.05; knowledge sharing: β=0.09, p>0.05) nor significant mediated effects via perceived similarity (speaking up: β=0.05, p>0.05; silence: β=0.03, 0>0.05; knowledge sharing: β=0.04, p>0.05). Therefore, hypothesis 3 is not supported.

## Discussion

This study investigates the relationships among generational diversity, perceived similarity, speaking up, silence and knowledge sharing in rural Chinese hospitals. In contrast to the theory-informed hypotheses formulated, this study finds no significant relationships between generational diversity and the other four variables (i.e., perceived similarity, speaking up, silence and knowledge sharing) and therefore appears to exclude significant direct and mediated effects of generational diversity on these variables.

Generational diversity is defined based on age groups and is therefore considered surface-level attribute.[19,20] Literature shows that surface-level similarity does not necessarily align with perceived similarity.[20,77] We attempted to establish the connection between generational diversity and perceived similarity via intergenerational differences as the latter two are related to deep-level attributes (e.g., values, attitudes and beliefs) but found non-significant relationship. Other determinants of perceived similarity exist as well, which may influence perceived similarity more strongly. For instance, Ott-Holland et al. demonstrate that gender plays a role in perceived similarity, and females perceive themselves to be more similar to others than do males.[78] H. Wang et al. found that local origin may impact perceived similarity.[16] People raised in the same area share the local culture, norms and social background and therefore are more likely to form similar values and beliefs. Of the respondents included in this study, 86.85% were born and raised in the areas where



For peer review only - http://bmjopen.bmj.com/site/about/guidelines.xhtml

their hospitals are located. An additional analysis, however, reveals that the composition of local and non-local employees is not significantly related to perceived similarity, nor to individual behaviours (Appendix 2). Therefore, more research is needed to understand the determinants of perceived similarity.

Our findings show that perceived similarity is positively associated with speaking up and knowledge sharing at both individual and team level. These findings confirm the similarity attraction theory, which posits such interactive behaviours to be associated with similarity.[25] The social identity theory[27] and the self-categorisation theory[28] provide further theoretical support for this finding. Similar people will categorise themselves as "ingroups", enhance their self-image and then be more willing to interact with each other. Conversely, individuals perceive dissimilar ones as "outgroups" and amplify the differences between "ingroups" and "outgroups", which may form intergroup conflicts and ultimately reduce the frequency of intergroup interactive behaviours. The evidence found and the theoretical support thus indicate that increasing the recruitment of healthcare workforce in rural Chinese hospitals (and other rural settings) to strengthen the quality of care and UHC may negatively influence speaking up and knowledge sharing and impede teamwork if it causes increased perceived dissimilarity.

Counter to our hypothesis, perceived similarity is positively related to silence at both individual and team level. The social identity theory[27] and the self-categorisation theory[28] may provide explanations for this finding. People consider similar team members to be part of their "ingroups", which will stimulate personal relationships. These personal relationships may subsequently lead them to refrain from commenting openly on the functioning of these "ingroup" members. As this study was conducted in rural China, the Chinese cultural values of "saving face" and "harmony" to promote team functioning may further amplify this mechanism.[79] This cultural perspective sheds a novel light on the common, perhaps Western, view that silence is harmful to team functioning in healthcare.[31] Furthermore, team members may be less likely to remain silent with dissimilar team members ("outgroups"), as perceived dissimilarity is often accompanied by dissatisfaction with others.[80,81]

In addition to the positive association between perceived similarity and both speaking up and silence, we somewhat surprisingly find a positive correlation between the latter two. This correlation may be explained by distinguishing various subtypes of speaking up and silence. Scientific literature has identified acquiescent speaking up/silence (expressing/withholding ideas based on resignation and low self-efficacy), defensive speaking up/silence (expressing/withholding ideas based on self-protection and fear) and prosocial speaking up/silence (expressing/withholding ideas based on benefiting the organisation and cooperation).[43] These different forms of speaking up and silence can explain why team members may or may not express constructive suggestions. For example, speaking up with the goal of benefiting the team while keeping silent to protect themselves and their "ingroups" on other occasions. Our findings from the rural Chinese context thus confirm that speaking up and silence are distinct constructs rather than a pair of opposite behaviours.[43,44] It is worthwhile to investigate subtypes of speaking up and silence, and their relationship with perceived similarity in future research on team functioning and performance.

In a nutshell, this study has provided insights into the influence diversity has on team functioning in rural Chinese hospitals. The findings suggest that rural hospitals' efforts to recruit a more generationally diverse healthcare workforce in recent years are not likely to have directly impacted team functioning or the perceived similarity. At the same time, our results reveal that perceived similarity is likely to enhance teamwork behaviour. Hence, the effects of the necessary workforce strengthening efforts on perceived diversity deserve continued management attention and support.



For peer review only - http://bmjopen.bmj.com/site/about/guidelines.xhtml

The benefits of similarity need to be balanced with the value of (generational) diversity in team functioning in rural Chinese hospitals, as also confirmed by evidence from Western countries.[5,6] There is qualitative evidence that older doctors appreciate the knowledge and energy brought by the younger generations.[16] Future qualitative and quantitative studies are needed to further explore the role of perceived similarity in team functioning in rural Chinese hospitals and the mechanisms by which hypothesised antecedents such as generational diversity impact perceived similarity. These mechanisms and antecedents may be context specific, and further research on the role of diversity in strengthening the rural health workforce in pursuit of SDG3 is therefore also called for.

### *Practical implications*

Our study shows that teamwork behaviour is associated with perceived similarity yet less so with generational diversity. This is relevant because the teamwork behaviours studied (i.e. speaking up, silence and knowledge sharing) are associated with team performance and quality of care. Thus, rural Chinese hospital management may leverage the benefits of perceived similarity for team functioning to improve the quality of care and UHC. At the same time, our findings indicate that increases in generational diversity, which may result from ongoing workforce strengthening efforts, will not negatively impact the studied teamwork behaviours in support of healthcare quality and achieving UHC. However, when recruitment efforts for workforce strengthening lead to greater perceived dissimilarity in teams, these dissimilarities need to be managed, for instance through team training, to avoid negative impacts on team functioning and, possibly, quality of care.

### *Strengths and Limitations*

This study provides insights into the relationship between generational diversity, perceived similarity and team functioning in rural Chinese hospitals based on a large survey study. Four Chinese county-level hospitals were included in the data collection. Although this resulted in a sufficient number of respondents, the selection of solely county-level hospitals may lead to a selection bias and may not depict a full picture of all rural Chinese hospitals. Moreover, as the number of people who received the survey link is unknown, the response rate is unclear.

Nevertheless, the four participating hospitals represent considerable geographical diversity with two of them being from distinct northern Chinese regions, and the other two from distinct southern Chinese regions. The sample size and variation of demographic characteristics and backgrounds are a strength of this study and ensures that the respondent sample represents the workforce in rural county-level hospitals and differs from national and provincial hospitals. This increases the generalisability to other rural county-level hospitals and at the same time limits the generalisability to other types of hospitals. This survey was constructed on the basis of validated measures, which strengthens the survey design. Nevertheless, all the variables were measured by the same respondents and via the same data collection method (survey), which may produce common source bias and common method bias. Lastly, the interpretation of our findings should take into account that the cross-sectional design does not allow claims about causality of the significant relationships between perceived similarity and the three teamwork behaviours studied.

## Conclusion



For peer review only - http://bmjopen.bmj.com/site/about/guidelines.xhtml

Perceived similarity is positively related to speaking up, silence and knowledge sharing at both individual and team level. Generational diversity has no significant relationships with perceived similarity nor with these three teamwork behaviours. Thus, if healthcare workforce strengthening increases generational diversity, this may not impact team behaviour and performance. However, explicit management efforts to mitigate the negative impact on team behaviour and care provision are called for if the healthcare workforce strengthening required for improving quality of care and UHC reduces perceived similarity in teams. More research is needed to explore the antecedents of (deep-level) perceived similarity and the interaction between perceived similarity and various forms of speaking up and silence.

**Contributors**

HW, MBS, JVW and JVDK designed the study. HW collected the data. HW and MBS analysed the data. HW drafted the manuscript. HW, MBS, JVW and JVDK revised and approved the manuscript. HW is the guarantor.

**Acknowledgement**

We thank Yun Zhang and Jing Zhou from the Health Human Resources Development Centre of the National Health Commission of China, and Yan'an Wang and Xiaoxiao Wan from the County Health Media for helping connect Chinese hospitals. We are also grateful for Wenxing Wang's participation in the translation/back-translation before data collection. Most importantly, we show our highest gratitude to the presidents and all the respondents from the 4 rural Chinese hospitals for their contributions to this study.

**Ethical statement**

The Research Ethics Review Committee of Erasmus School of Health Policy and Management, Erasmus University Rotterdam approved this study (No. ETH2122-0807). Consent was obtained from each respondent before the data collection.

**Conflicts of interest**

The authors declare that this study was conducted without any conflicts of interest.

**Funding**

This study was supported by China Scholarship Council (Grand number: 201906160092). This funder has no role in the study design, data collection, data analysis and writing the manuscript.

**Data availability statement**

The data that support the findings of this study are available from the corresponding author upon reasonable request.



For peer review only - http://bmjopen.bmj.com/site/about/guidelines.xhtml

**Figure legend**

Figure 1. Theoretical Model

**References**

1. World Health Organization. Targets of Sustainable Development Goal. Available: https://www.who.int/data/gho/data/themes/sustainable-development-goals [Accessed 13 Feb 2024].

2. Lehmann U, Dieleman M, Martineau T. Staffing remote rural areas in middle-and low-income countries: a literature review of attraction and retention. *BMC Health Serv Res* 2008;**8**:19. doi:10.1186/1472-6963-8-19

3. Maffioli EM, Rocha TAH, Vivas G, *et al*. Addressing inequalities in medical workforce distribution: evidence from a quasi-experimental study in Brazil. *BMJ Glob Health* 2019;**4**:e001827. doi:10.1136/bmjgh-2019-001827

4. Sheikh K, Josyula LK, Zhang X, *et al*. Governing the mixed health workforce: learning from Asian experiences. *BMJ Glob Health* 2017;**2**:e000267. doi:10.1136/bmjgh-2016-000267

5. Gomez LE, Bernet P. Diversity improves performance and outcomes. *J Natl Med Assoc* 2019;**111**:383-92. doi:10.1016/j.jnma.2019.01.006

6. Guglielminotti J, Samari G, Friedman AM, *et al*. Nurse workforce diversity and reduced risk of severe adverse maternal outcomes. *Am J Obstet Gynecol MFM* 2022;**4**:100689. doi:10.1016/j.ajogmf.2022.100689

7. Spinks N, Moore C. The changing workforce, workplace and nature of work: implications for health human resource management. *Nurs Leadersh* 2007;**20**:26-41. doi:10.12927/cjnl.2007.19286

8. Lyons S, Kuron L. Generational differences in the workplace: a review of the evidence and directions for future research. *J Organ Behav* 2014;**35**:S139-57. doi: 10.1002/job.1913

9. Woodward I, Vongswasdi P, More E. Generational Diversity at Work: a Systematic Review of the Research. INSEAD Working Paper No. 2015/48/OB, 2015. doi:10.2139/ssrn.2630650

10. Bachus V, Dishman L, Fick JW. Improving workforce experiences at United States Federally Qualified Health Centers: exploring the perceived impact of generational diversity on employee engagement. *Patient Exp J* 2022;**9**:17-30. doi:10.35680/2372-0247.1715

11. Hapsari C, Stoffers J, Gunawan A. The influence of generational diversity management and leader–member exchange on innovative work behaviors mediated by employee engagement. *J Asia Pac Bus* 2019;**20**:125-39. doi:10.1080/10599231.2019.1610684

12. World Bank. GDP growth (annual %). World Bank. Available: https://data.worldbank.org/indicator/NY.GDP.MKTP.KD.ZG [Accessed 13 Feb 2024].

13. Yi X, Ribbens B, Morgan CN. Generational differences in China: career implications. *Career Dev Int* 2010;**15**:601-20. doi:10.1108/13620431011084439



For peer review only - http://bmjopen.bmj.com/site/about/guidelines.xhtml

14. Cennamo L, Gardner D. Generational differences in work values, outcomes and person-organisation values fit. *J Manag Psychol* 2008;**23**:891-906. doi:10.1108/02683940810904385

15. Shragay D, Tziner A. The generational effect on the relationship between job involvement, work satisfaction, and organizational citizenship behavior. *J Work Organ Psychol* 2011;**27**:143-57. doi:10.5093/tr2011v27n2a6

16. Wang H, van Wijngaarden J, Buljac-Samardzic M, *et al*. Factors and interventions determining the functioning of health care teams in county-level hospitals in less affluent areas of China: a qualitative study. *Front Public Health* 2023;**11**:1082070. doi:10.3389/fpubh.2023.1082070

17. National Bureau of Statistics of China. Statistical communique of the People's Republic of China on the 2022 national economic and social development. National Bureau of Statistics of China; 2023. Available: http://www.stats.gov.cn/english/PressRelease/202302/t20230227_1918979.html [Accessed 13 Feb 2024].

18. Van de Klundert J, van Dongen-van den Broek J, Yesuf EM, *et al*. 'We are planning to leave, all of us'—a realist study of mechanisms explaining healthcare employee turnover in rural Ethiopia. *Hum Resour Health* 2018;**16**:37. doi:10.1186/s12960-018-0301-0

19. Van Emmerik IH, Brenninkmeijer V. Deep-level similarity and group social capital: associations with team functioning. *Small Group Res* 2009;**40**:650-69. doi:10.1177/1046496409346550

20. Williams HM, Parker SK, Turner N. Perceived dissimilarity and perspective taking within work teams. *Group Organ Manag* 2007;**32**:569-97. doi:10.1177/1059601106293769

21. Becker KL, Richards MB, Stollings J. Better together? Examining benefits and tensions of generational diversity and team performance. *J Int Relatsh*, 2022;**20**:442-63. doi:10.1080/15350770.2020.1837708

22. Tolbize A. *Generational differences in the workplace*. Minneapolis, MN: Research and Training Center on Community Living, 2008.

23. Jansen AE, Searle BJ. Diverse effects of team diversity: a review and framework of surface and deep-level diversity. *Pers Rev* 2021;**50**:1838-53. doi:10.1108/PR-12-2019-0664

24. Shemla M, Meyer B, Greer L, *et al*. A review of perceived diversity in teams: does how members perceive their team's composition affect team processes and outcomes?. *J Organ Behav* 2016;37:S89-106. doi:10.1002/job.1957

25. Byrne DE. *The attraction paradigm (Ser. Personality and psychopathology, 11)*. New York: Academic Press, 1971.

26. Mehra A, Kilduff M, Brass DJ. At the margins: a distinctiveness approach to the social identity and social networks of underrepresented groups. *Acad Manag J* 1998;**41**:441-52. doi:10.5465/257083

27. Tajfel H. Social identity and intergroup behaviour. *Soc Sci Inf* 1974;**13**:65-93. doi:10.1177/053901847401300204

28. Turner JC. Social categorization and the self-concept: a social cognitive theory of group behavior. In: Postmes T, Branscombe NR, editors. *Rediscovering Social Identity*. Psychology Press, 2010:243-72.



For peer review only - http://bmjopen.bmj.com/site/about/guidelines.xhtml

29. Kolbe M, Burtscher MJ, Wacker J, *et al*. Speaking up is related to better team performance in simulated anesthesia inductions: an observational study. *Anesth Analg* 2012;**115**:1099-108. doi:10.1213/ANE.0b013e318269cd32

30. Okuyama A, Wagner C, Bijnen B. Speaking up for patient safety by hospital-based health care professionals: a literature review. *BMC Health Serv Res* 2014;**14**:61. doi:10.1186/1472-6963-14-61

31. Henriksen K, Dayton E. Organizational silence and hidden threats to patient safety. *Health Serv Res* 2006;**41**:1539-54. doi:10.1111/j.1475-6773.2006.00564.x

32. Chang CW, Huang HC, Chiang CY, *et al*. Social capital and knowledge sharing: effects on patient safety. *J Adv Nurs* 2012;**68**:1793-803. doi:10.1111/j.1365-2648.2011.05871.x

33. Karamitri I, Talias MA, Bellali T. Knowledge management practices in healthcare settings: a systematic review. *Int J Health Plan Manag* 2017;**32**:4-18. doi:10.1002/hpm.2303

34. Kessel M, Kratzer J, Schultz C. Psychological safety, knowledge sharing, and creative performance in healthcare teams. *Creat Innov Manag* 2012;**21**:147-57. doi:10.1111/j.1467-8691.2012.00635.x

35. Moore S, Grunberg L, Krause AJ. Generational differences in workplace expectations: a comparison of production and professional workers. *Curr Psychol* 2015;**34**:346-62. doi:10.1007/s12144-014-9261-2

36. Liang J, Xu Y. Second-hand clothing consumption: a generational cohort analysis of the Chinese market. *Int J Consum Stud* 2018;**42**:120-30. doi:10.1111/ijcs.12393

37. Yi X, Ribbens B, Fu L, *et al*. Variation in career and workplace attitudes by generation, gender, and culture differences in career perceptions in the United States and China. *Employee Relat* 2015;**37**:66-82. doi:10.1108/ER-01-2014-0005

38. Zuo B, Lai Z. The effect of housing wealth on tourism consumption in China: age and generation cohort comparisons. *Tour Econ* 2020;**26**:211-32. doi:10.1177/1354816619861843

39. Morry MM, Kito MIE, Ortiz L. The attraction–similarity model and dating couples: projection, perceived similarity, and psychological benefits. *Pers Relatsh* 2011;**18**:125-43. doi:10.1111/j.1475-6811.2010.01293.x

40. Jones JS, Murray SR, Tapp SR. Generational differences in the workplace. *J Bus Divers* 2018;**18**:88-97. doi:10.33423/jbd.v18i2.528

41. Mowbray PK, Wilkinson A, Tse HH. An integrative review of employee voice: identifying a common conceptualization and research agenda. *Int J Manag Rev* 2015;**17**:382-400. doi:10.1111/ijmr.12045

42. Premeaux SF, Bedeian AG. Breaking the silence: the moderating effects of self-monitoring in predicting speaking up in the workplace. *J Manag Stud* 2003;**40**:1537-62. doi:10.1111/1467-6486.00390

43. Van Dyne L, Ang S, Botero IC. Conceptualizing employee silence and employee voice as multidimensional constructs. *J Manag Stud* 2003;**40**:1359-92. doi:10.1111/1467-6486.00384

44. Sherf EN, Parke MR, Isaakyan S. Distinguishing voice and silence at work: unique relationships with perceived impact, psychological safety, and burnout. *Acad Manag J* 2021;**64**:114-48. doi:10.5465/amj.2018.1428



For peer review only - http://bmjopen.bmj.com/site/about/guidelines.xhtml

45. Knoll M, Redman T. Does the presence of voice imply the absence of silence? The necessity to consider employees' affective attachment and job engagement. *Hum Resour Manag* 2016;**55**:829-44. doi:10.1002/hrm.21744

46. Qazi W, Raza SA, Khan KA. The contradiction between self-protection and self-presentation on knowledge sharing behaviour: evidence from higher education students in Pakistan. *Int J Knowl Learn* 2020;**13**:246-71. doi:10.1504/IJKL.2020.109910

47. Yi J. A measure of knowledge sharing behavior: scale development and validation. *Knowledge Manag Res Pract* 2009;**7**:65-81. doi:10.1057/kmrp.2008.36

48. Lainidi O, Jendeby MK, Montgomery A, *et al*. An integrative systematic review of employee silence and voice in healthcare: what are we really measuring?. *Front Psychiatry* 2023;**14**:1111579. doi:10.3389/fpsyt.2023.1111579

49. Lee SE, Choi J, Lee H, *et al*. Factors influencing nurses' willingness to speak up regarding patient safety in East Asia: a systematic review. *Risk Manag Healthc Policy* 2021;**14**:1053-63. doi:10.2147/RMHP.S297349

50. Lu J, Xie X. Research on employee silence behavior: a Review based on Chinese family enterprise. *Asian Soc Sci* 2013;**9**:47-52. doi:10.5539/ass.v9n17p47

51. Wang S, Noe RA. Knowledge sharing: a review and directions for future research. *Hum Resour Manag Rev* 2010;**20**:115-31. doi:10.1016/j.hrmr.2009.10.001

52. Deniz S, Çimen M. The mediating role of work alienation in the effect of workplace ostracism on employee voice. *Hospital Topics* 2022. doi:10.1080/00185868.2022.2116375

53. Imran MK, Fatima T, Sarwar A, *et al*. Will I speak up or remain silent? Workplace ostracism and employee performance based on self-control perspective. *J Soc Psychol* 2023;**163**:107-25. doi:10.1080/00224545.2021.1967843

54. Takhsha M, Barahimi N, Adelpanah A, *et al*. The effect of workplace ostracism on knowledge sharing: the mediating role of organization-based self-esteem and organizational silence. *J Workplace Learn* 2020;**32**:417-35. doi:10.1108/JWL-07-2019-0088

55. Wang GH, Li JH, Liu H, *et al*. The association between workplace ostracism and knowledge-sharing behaviors among Chinese university teachers: the chain mediating model of job burnout and job satisfaction. *Front Psychol* 2023;14:1030043. doi:10.3389/fpsyg.2023.1030043

56. Gkorezis P, Panagiotou M, Theodorou M. Workplace ostracism and employee silence in nursing: the mediating role of organizational identification. *J Adv Nurs* 2016;**72**:2381-88. doi:10.1111/jan.12992

57. Bhatti SH, Hussain M, Santoro G, *et al*. The impact of organizational ostracism on knowledge hiding: analysing the sequential mediating role of efficacy needs and psychological distress. *J Knowl Manag* 2023;**27**:485-505. doi:10.1108/JKM-03-2021-0223

58. Zhao H, Xia Q, He P, *et al*. Workplace ostracism and knowledge hiding in service organizations. *Int J Hosp Manag* 2016;**59**:84-94. doi:10.1016/j.ijhm.2016.09.009

59. Blau PM. *Inequality and heterogeneity: A primitive theory of social structure*. New York: Free Press, 1977.



For peer review only - http://bmjopen.bmj.com/site/about/guidelines.xhtml

60. Liden RC, Wayne SJ, Stilwell D. A longitudinal study on the early development of leader-member exchanges. *J Appl Psychol* 1993;**78**:662-74. doi: 10.1037/0021-9010.78.4.662

61. Van Dyne L, LePine JA. Helping and voice extra-role behaviors: evidence of construct and predictive validity. *Acad Manag J* 1998;**41**:108-19. doi:10.5465/256902

62. Morrison EW, Wheeler-Smith SL, Kamdar D. Speaking up in groups: a cross-level study of group voice climate and voice. *J Appl Psychol* 2011;**96**:183-91. doi:10.1037/a0020744

63. Detert JR, Edmondson AC. Implicit voice theories: taken-for-granted rules of self-censorship at work. *Acad Manag J* 2022;**54**:461-88. doi:10.5465/amj.2011.61967925

64. Guenter H, Schreurs B, van Emmerik IH, *et al*. What does it take to break the silence in teams: authentic leadership and/or proactive followership?. *Appl Psychol* 2017;**66**:49-77. doi:10.1111/apps.12076

65. Mignonac K, Herrbach O, Serrano Archimi C, *et al*. Navigating ambivalence: perceived organizational prestige–support discrepancy and its relation to employee cynicism and silence. *J Manag Stud* 2018;**55**:837-72. doi:10.1111/joms.12330

66. Pittino D, Martínez AB, Chirico F, *et al*. Psychological ownership, knowledge sharing and entrepreneurial orientation in family firms: the moderating role of governance heterogeneity. *J Bus Res* 2018;**84**:312-26. doi:10.1016/j.jbusres.2017.08.014

67. Bartol KM, Liu W, Zeng X, *et al*. Social exchange and knowledge sharing among knowledge workers: the moderating role of perceived job security. *Manag Organ Rev* 2009;**5**:223-40. doi:10.1111/j.1740-8784.2009.00146.x

68. Dong Y, Bartol KM, Zhang ZX, *et al*. Enhancing employee creativity via individual skill development and team knowledge sharing: Influences of dual-focused transformational leadership. *J Organ Behav* 2017;**38**:439-58. doi:10.1002/job.2134

69. LePine JA, Van Dyne L. Predicting voice behavior in work groups. *J Appl Psychol* 1998;**83**:853-68. doi:10.1037/0021-9010.83.6.853

70. Post C. When is female leadership an advantage? Coordination requirements, team cohesion, and team interaction norms. *J Organ Behav* 2015;**36**:1153-75. doi:10.1002/job.2031

71. Behling O, Law KS. *Translating questionnaires and other research instruments: problems and solutions*. Thousand Oaks, CA: Sage, 2000. doi:10.4135/9781412986373

72. Bell A, Fairbrother M, Jones K. Fixed and random effects models: making an informed choice. *Qual Quant* 2019;**53**:1051-74. doi:10.1007/s11135-018-0802-x

73. Rockwood NJ. *Advancing the formulation and testing of multilevel mediation and moderated mediation models (Master's thesis)*. The Ohio State University, Columbus, OH, 2017.

74. Sharma A, Minh Duc NT, Luu Lam Thang T, *et al*. A consensus-based checklist for reporting of survey studies (CROSS). *J Gen Intern Med* 2021;36:3179-87. doi: 10.1007/s11606-021-06737-1



For peer review only - http://bmjopen.bmj.com/site/about/guidelines.xhtml

75. National Health Commission. The statistical bulletin of the development of health services in China in 2022. National Health Commission; 2023. Available: http://www.nhc.gov.cn/cms-search/downFiles/9b3fddc4703d4c9d9ad399bcca089f03.pdf [Accessed 13 Feb 2024].

76. Kim JH. Multicollinearity and misleading statistical results. *Korean J Anesthesiol* 2019;**72**:558-69. doi:10.4097/kja.19087

77. Jehn KA, Northcraft GB, Neale MA. Why differences make a difference: A field study of diversity, conflict and performance in workgroups. *Adm Sci Q* 1999;**44**:741-63. doi:10.2307/2667054

78. Ott-Holland CJ, Huang JL, Ryan AM, *et al*. The effects of culture and gender on perceived self-other similarity in personality. *J Res Personal* 2014;**53**:13-21. doi:10.1016/j.jrp.2014.07.010

79. Cardon PW, Scott JC. Chinese business face: communication behaviors and teaching approaches. *Bus Commun Q* 2003;**66**:9-22. doi:10.1177/108056990306600402

80. Becker JC, Tausch N. When group memberships are negative: the concept, measurement, and behavioral implications of psychological disidentification. *Self Identity* 2014;**13**:294-321. doi:10.1080/15298868.2013.819991

81. Sultana MS, Niu X, Hossain MS. How dissimilarity attributes at restaurants trigger negative emotions and associated behavioral intentions: the role of attribute performance. *J Hosp and Tour Insights* 2023;6:2199-221. doi:10.1108/JHTI-03-2022-0115

For peer review only

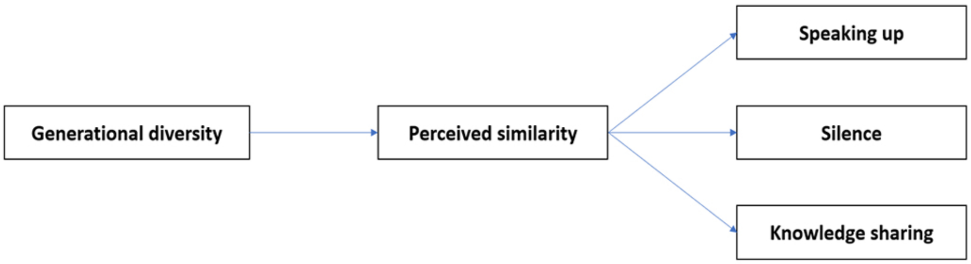


Figure 1. Theoretical Model

90x90mm (300 x 300 DPI)

## Appendix 1. Measures for perceived similarity, speaking up, silence and knowledge sharing

***Perceived similarity*[20,60]**

1. Team members handle problems in a similar way.

2. Team members think alike in terms of coming up with a similar solution.

3. Team members analyse problems in a similar way.

4. Team members see things in much the same way.

5. Team members are similar in terms of outlook and values.

6. Team members are alike in a number of areas.

***Speaking up*[61,62]**

1. I develop and make recommendations concerning issues that affect this team.

2. I speak up and encourage others in this team to get involved in issues that affect this team.

3. I communicate my opinions about work issues to others in this team even if my opinion is different and

4. I keep well informed about issues where my opinion might be useful to this team.

5. I get involved in issues that affect the quality of work life here in this team.

6. I speak up in this team with ideas for new projects or changes in procedures.

***Silence*[63-65]**

1. I withhold ideas from the team leader for changing inefficient work policies.

2. I keep ideas for developing new products or services to myself.

3. I do not speak up about difficulties caused by the way the team leader and the team members interact.

4. I keep quiet in team meetings about problems with daily routines that hamper performance.

5. I withhold thoughts about improving patients' experiences with us.

***Knowledge sharing*[66,67]**

1. Team members share information that can be helpful to the team.

2. Team members keep information flow high to increase team effectiveness.

3. Team members seek helpful information to be shared within the team.

For peer review only

4. Team members share expertise to help resolve the team's problems.

5. Team members collectively offer innovative ideas that can benefit the team.

For peer review only

**Appendix 2.** *Additional multilevel mediation analysis (Diversity in the composition of local and non-local healthcare professionals)*

| | Perceived similarity | Speak up | Silence | Knowledge sharing |
|---|---|---|---|---|
| ***Fixed effects*** | | | | |
| **Within-group (Level 1)** | | | | |
| Intercept | 5.54** | 1.76** | 1.62** | 2.39** |
| **Perceived similarity** | - | 0.56** | 0.39** | 0.54** |
| Gender | 0.23 | 0.04 | -0.31 | 0.30** |
| Team tenure | -0.00 | 0.00 | 0.01 | -0.01 |
| **Between-group (Level 2)** | | | | |
| **Diversity in composition** | -0.22 | 0.08 | 0.50 | 0.16 |
| **Perceived similarity** | - | 0.70** | 0.40** | 0.60** |
| Gender | 0.25 | -0.14 | 0.15 | 0.16 |
| Team tenure | 0.01 | 0.01 | 0.03 | 0.00 |
| Team size | -0.00 | -0.00 | -0.00 | -0.00 |
| **Indirect effect (Mediation)** | - | -0.16 | -0.09 | -0.14 |
| ***Random effects (Variance)*** | | | | |
| Intercept | 0.19** | 0.03 | 0.40** | 0.04 |
| Slope | - | 0.12** | 0.04 | 0.11** |

**: p<0.05; **: p<0.01*

For peer review only
